# SARS-CoV-2 binds platelet ACE2 to enhance thrombosis in COVID-19

**DOI:** 10.1186/s13045-020-00954-7

**Published:** 2020-09-04

**Authors:** Si Zhang, Yangyang Liu, Xiaofang Wang, Li Yang, Haishan Li, Yuyan Wang, Mengduan Liu, Xiaoyan Zhao, Youhua Xie, Yan Yang, Shenghui Zhang, Zhichao Fan, Jianzeng Dong, Zhenghong Yuan, Zhongren Ding, Yi Zhang, Liang Hu

**Affiliations:** 1 Department of Cardiology, the First Affiliated Hospital of Zhengzhou University, Cardiovascular Institute of Zhengzhou University, Zhengzhou, 450052 China; 2 Department of Biochemistry and Molecular Biology, NHC Key Laboratory of Glycoconjugates Research, School of Basic Medical Sciences, Fudan University, Shanghai, 200032 China; 3 Biotherapy Center, the First Affiliated Hospital of Zhengzhou University, Zhengzhou, 450052 China; 4 Department of Emergency, Affiliated Hospital of Anhui Medical University, Hefei, China; 5 Key Laboratory of Medical Molecular Virology (MOE/NHC/CAMS), and Department of Medical Microbiology and Parasitology, School of Basic Medical Sciences, Shanghai Medical College, Fudan University, Shanghai, China; 6 School of Public Health (Shenzhen), Sun Yat-sen University, Guangzhou, China; 7 Department of Hematology, Wenzhou Key Laboratory of Hematology, The First Affiliated Hospital of Wenzhou Medical University, Wenzhou, China; 8 Department of Immunology, School of Medicine, UConn Health, Farmington, CT 06030 USA

**Keywords:** COVID-19, Thrombosis, Platelet activation, ACE2, TMPRSS2

## Abstract

**Background:**

Critically ill patients diagnosed with COVID-19 may develop a pro-thrombotic state that places them at a dramatically increased lethal risk. Although platelet activation is critical for thrombosis and is responsible for the thrombotic events and cardiovascular complications, the role of platelets in the pathogenesis of COVID-19 remains unclear.

**Methods:**

Using platelets from healthy volunteers, non-COVID-19 and COVID-19 patients, as well as wild-type and hACE2 transgenic mice, we evaluated the changes in platelet and coagulation parameters in COVID-19 patients. We investigated ACE2 expression and direct effect of SARS-CoV-2 virus on platelets by RT-PCR, flow cytometry, Western blot, immunofluorescence, and platelet functional studies in vitro, FeCl_3_-induced thrombus formation in vivo, and thrombus formation under flow conditions ex vivo.

**Results:**

We demonstrated that COVID-19 patients present with increased mean platelet volume (MPV) and platelet hyperactivity, which correlated with a decrease in overall platelet count. Detectable SARS-CoV-2 RNA in the blood stream was associated with platelet hyperactivity in critically ill patients. Platelets expressed ACE2, a host cell receptor for SARS-CoV-2, and TMPRSS2, a serine protease for Spike protein priming. SARS-CoV-2 and its Spike protein directly enhanced platelet activation such as platelet aggregation, PAC-1 binding, CD62P expression, α granule secretion, dense granule release, platelet spreading, and clot retraction in vitro, and thereby Spike protein enhanced thrombosis formation in wild-type mice transfused with hACE2 transgenic platelets, but this was not observed in animals transfused with wild-type platelets in vivo. Further, we provided evidence suggesting that the MAPK pathway, downstream of ACE2, mediates the potentiating role of SARS-CoV-2 on platelet activation, and that platelet ACE2 expression decreases following SARS-COV-2 stimulation. SARS-CoV-2 and its Spike protein directly stimulated platelets to facilitate the release of coagulation factors, the secretion of inflammatory factors, and the formation of leukocyte–platelet aggregates. Recombinant human ACE2 protein and anti-Spike monoclonal antibody could inhibit SARS-CoV-2 Spike protein-induced platelet activation.

**Conclusions:**

Our findings uncovered a novel function of SARS-CoV-2 on platelet activation via binding of Spike to ACE2. SARS-CoV-2-induced platelet activation may participate in thrombus formation and inflammatory responses in COVID-19 patients.

## Background

The COVID-19 pandemic has become a serious public health crisis worldwide since December 2019 [1]. COVID-19 has been linked to a number of critical cardiovascular complications [2, 3], and even individuals without a history of cardiovascular disease are at risk of cardiovascular complications [4]. Patients with severe COVID-19 commonly experience thrombotic disorders, sepsis, and disseminated intravascular coagulation (DIC), and these conditions have been closely linked to higher mortality rates [1, 5, 6]. Large-scale studies have revealed that 18.8% to 36.2% of patients [7, 8] present with thrombocytopenia on admission. In addition, the cumulative incidence of thrombotic complications for COVID-19 patients in the ICU was 31%, while only 1.3% of non-COVID-19 ICU patients experience thrombotic complications [9]. Although the evidence supports a link between COVID-19 and the development of a hypercoagulable state, the underlying mechanisms for this association remain elusive.

Platelets are known for their critical contributions to thrombosis and hemostasis [10–12]. During infection, activated platelets adhere to the sub-endothelium, and their hyperactivity results in thrombus formation, leading to arterial ischemia and even pulmonary embolisms. Many viruses, including human immunodeficiency virus (HIV), hepatitis C virus (HCV), influenza virus, Ebola, and Dengue virus (DV), can directly lead to platelet hyperactivity [13–16]. Influenza virus directly activates platelets and triggers uncontrolled coagulation cascades and consequent lung injury [17–19]. Although COVID-19 is a respiratory disease, SARS-CoV-2 RNA can be detected in the blood and used as an indicator of disease severity [20, 21]. Currently, whether the COVID-19 virus can directly activate platelets, and therefore promote its pro-thrombotic function remains unclear.

The pathogen causing COVID-19 is severe acute respiratory syndrome coronavirus 2 (SARS-CoV-2), an enveloped RNA virus, and the seventh member of the human coronavirus family [22]. SARS-CoV-2 uses its Spike protein to enter host cells by binding to angiotensin-converting enzyme 2 (ACE2) on the host cell membrane [23–26]. Meanwhile, transmembrane protease serine 2 (TMPRSS2), a serine protease, proteolytically cleaves and activates the Spike protein to facilitate SARS-CoV-2 virus-cell membrane fusions. Although the Spike protein from SARS-CoV-2 has been reported to bind to ACE2 and manipulate various cell functions [27–31], it has not been addressed if platelets express ACE2 and TMPRSS2.

Here, we report that platelets from COVID-19 patients are hyperactive, and demonstrate, for the first time, that platelets express ACE2 and TMPRSS2. SARS-CoV-2 and its Spike protein directly bind platelet ACE2 and enhance platelet activation in vitro. The Spike protein also potentiates thrombus formation in vivo. Moreover, we were able to demonstrate that SARS-CoV-2 and its Spike protein directly stimulate platelets resulting in coagulation factor release, inflammatory cytokine secretion, and leukocyte–platelet aggregates (LPAs) formation. Finally, we provide evidence that treatment with recombinant human ACE2 protein and an anti-Spike monoclonal antibody can reverse SARS-CoV-2 Spike protein-induced platelet activation.

## Methods

Detailed materials and methods are described in Additional file 1: Expanded Materials and Methods.

### Study design

For this study, we recruited COVID-19 patients admitted to the First Affiliated Hospital of Zhengzhou University, Henan Province, China and three centers of the Affiliated Hospital of Anhui Medical University, Anhui Province, China between January 10th and March 18th 2020. The detailed study design, including the inclusion and exclusion criteria as well as laboratory data collection, is described in Additional file 1: Expanded Materials and Methods. This study was approved by the Ethics Committee of the First Affiliated Hospital of Zhengzhou University (2020-KY-121) and the Ethics Committee of Anhui Medical University (2020-AH-114) and complied with the Declaration of Helsinki and good clinical practice guidelines. All participants provided written informed consent.

### Materials

Detailed descriptions of the reagents, commercial ELISA kits, and viral RNA detection in the blood are provided in Additional file 1: Expanded Materials and Methods. The primary antibodies and dilutions used in this study are listed in Additional file 1: Online Table 1.

### Washed platelets and peripheral blood mononuclear cells isolation

Platelets and peripheral blood mononuclear cells (PBMCs) were prepared [32] as detailed in Additional file 1: Expanded Materials and Methods. All experiments using human subjects were performed in accordance with the Declaration of Helsinki and approved by the Ethics Committee of Zhengzhou University.

### Platelet functional studies

Washed platelet aggregation and secretion in response to thrombin (0.025 U/mL) or collagen (0.6 μg/mL) as well as washed platelet aggregation in response to ADP (5 μM with 10 μg/mL fibrinogen) were measured as previously described [33]. Spreading and clot retraction were measured as previously described [34, 35] and detailed in Additional file 1: Expanded Materials and Methods. To explore the effects of SARS-CoV-2 and its Spike protein on platelet function, SARS-CoV-2 (1×10^5^ PFU [36]) or Spike protein (2 μg/mL) was added to platelets for the indicated times before agonist-induced stimulation [37–39].

### Cell culture

The human colon cell line Caco-2, the human lung cell line Calu-3, the human prostate cell line PC-3, and the human cervical carcinoma cell line HeLa were all cultured in the appropriate medium detailed in Additional file 1: Expanded Materials and Methods. All cells were collected and processed for RNA and protein extraction.

### SARS-CoV-2 virus preparation and incubation

SARS-CoV-2 virus was isolated from a COVID-19 patient in Shanghai (GenBank accession No. MT121215) and propagated in Vero E6 cells [23]. The supernatant from the SARS-CoV-2 infected cells was aliquoted and stored at − 80 °C until use. The supernatant from mock-infected cells was used as a control. For the platelet functional studies, around 300 μL of platelets (2 × 10^8^/mL) were incubated with 1 × 10^5^ PFU SARS-CoV-2 in Tyrode’s buffer. The infectious titers have been used in other cells [36]. All experiments involving live SARS-CoV-2 virus were performed in a biosafety level-3 (BLS-3) laboratory.

### Reverse transcription polymerase chain reaction

Total RNA was isolated from different cells and 1 μg of RNA was reverse transcribed to cDNA using an RNA isolation kit and polymerase chain reaction (RT-PCR) kit (TaKaRa, Japan), respectively [35]. PCR reactions were performed using specific primers (Additional file 1: Online Table 2).

### Flow cytometry analysis

Flow cytometry was used to evaluate platelet activity in COVID-19 patients, non-COVID-19 patients and healthy volunteers, and to evaluate platelet activity and ACE2 and TMPRSS2 expression in SARS-CoV-2 virus or Spike protein treated healthy platelets and leukocyte-platelet aggregates. All of these assays were completed using the previously described methods [23, 40, 41], and detailed in Additional file 1: Expanded Materials and Methods. The primary antibodies and dilutions used in these assays are listed in Additional file 1: Online Table 1.

### Electron microscopy

#### Sample preparation [42]

Platelet samples were incubated with SARS-CoV-2 and submitted to scanning electron microscopy (SEM) and transmission electron microscopy (TEM) as previously described. Briefly, platelets were placed in 6-well plates and incubated with 1 × 10^5^ PFU SARS-CoV-2 in Tyrode’s buffer under constant rotation for 30 min or 3 h at 37 °C. Platelets were then washed three times with PBS to remove virus and subjected to SEM and TEM as described previously [43], and detailed in the Additional file 1: Expanded Materials and Methods.

### Co-immunoprecipitation

Washed platelets subjected to different treatments were lysed with equal volumes of chilled 2× NP-40 lysis buffer (100 mM Tris-HCl pH 7.4, 300 mM NaCl, 2 mM NaF, 2% NP-40, 2 mM EDTA, and 2× protease and phosphatase inhibitor solution) on ice for 30 min. The supernatants were then precleared using protein A/G agarose beads for 3 h at 4 °C and then centrifuged. Immunoprecipitation was carried out using an anti-phospho-Ser/Thr antibody and then incubated with protein A/G-agarose beads overnight on a rocker at 4 °C. The beads were then harvested and rinsed 3 times with 1× NP-40 lysis buffer. Bead-captured ACE2 was detected by immunoblot and the primary antibodies and dilutions used in these assays are listed in Additional file 1: Online Table 1.

### Confocal microscopy

Platelets were attached to poly-l-Lysine-coated coverslips and fixed with precooled methanol and then blocked with BSA in PBS. After incubation with the primary antibodies and appropriate secondary antibodies, confocal images were captured using a laser-scanning confocal microscope as previously described [44] and detailed in Additional file 1: Expanded Materials and Methods. The primary antibodies and dilutions used in these assays are listed in Additional file 1: Online Table 1.

### ELISA assays

PF4, TNF-α, IL-8, IL-1β Factor V, and Factor XIII concentrations were determined using commercial ELISA kits according to the manufacturer’s instructions. More information is provided in Additional file 1: Expanded Materials and Methods.

### Animal studies

Wild-type C57BL/6 mice and hACE2 transgenic mice (Cat No. T037657) were purchased from Jiangsu Gempharmatech, China. All animal procedures were carried out in accordance with the ethical approval granted by the Ethics Committee of Zhengzhou University. Mouse platelets were prepared as described previously [35]. Adoptive platelet transfusions were performed based on previous reports [45]. More information is provided in Additional file 1: Expanded Materials and Methods.

### FeCl_3_-induced thrombosis formation in mouse mesenteric arterioles

Intravital microscopy of FeCl_3_-injured thrombus formation in mouse mesenteric arterioles was performed as previously described with minor modifications [34, 46, 47]. SARS-CoV-2 Spike protein (200 μg/kg) was injected intravenously into wild-type mice according to a previously reported method with minor modifications [30]. More information is provided in Additional file 1: Expanded Materials and Methods.

### Thrombus formation under flow conditions ex vivo

The flow chamber assay was prepared as described previously with minor modification [44, 48], and detailed in Additional file 1: Expanded Materials and Methods.

### Statistical analysis

Given the inherent differences between patients, a series of propensity score analyses were performed for the following variables: age, sex, history of smoking, hypertension, diabetes mellitus, hypercholesterolemia, stroke, and COPD. When matched with the healthy group, variables included age, sex, and history of smoking; when matched with other groups, variables included age, sex, history of smoking, hypertension, diabetes mellitus, hypercholesterolemia, stroke, and COPD. The propensity scores were estimated using a logit model. Matching was conducted using 1:1 or 1:2 nearest neighbor methods with a caliper width of 0.25*SDs in the logit propensity score, which yielded 28 severe and critically severe COVID-19 patients matched with 56 healthy subjects, 37 severe and critically severe COVID-19 patients matched with 37 non-COVID-19 patients, 29 severe and critically severe COVID-19 patients matched with 58 mild and moderate COVID-19 patients, 64 mild and moderate COVID-19 patients matched with 64 healthy subjects, and 59 non-COVID-19 subjects matched with 115 mild and moderate subjects. All matching analyses were performed using R software (version 3.6.0).

Continuous variables are expressed as the mean ± standard deviation of the mean (SD) or median (interquartile range was defined as the difference between twenty-fifth and seventy-fifth centiles) depending on data distribution, and compared using an unpaired Student’s *t* test or Mann-Whitney *U* test, as appropriate. Categorical variables were expressed as numbers and percentages and compared using the Chi-square test or Fisher’s exact test. All data were evaluated for normality (Kolmogorov-Smirnov) and subjected to the Bartlett’s test for homogeneity of group variances prior to statistical analysis. Group comparisons were made using one-way ANOVA, followed by Tukey’s post hoc analysis or Kruskal-Wallis test with Bonferroni correction. Pearson’s correlation analysis was used to investigate the relationships between two variables. A *P* value of less than 0.05 was considered statistically significant. Statistical analyses were performed using SPSS (version 21.0) and GraphPad Prism (7.0).

## Results

### Increased platelet activation in COVID-19

Our study population comprised 201 healthy volunteers and 589 patients suspected of having COVID-19. Of these 589 patients, 422 were identified as SARS-CoV-2 infected, while the remaining 167 patients were identified as non-SARS-CoV-2 infected. We excluded 35 healthy volunteers, 107 non-COVID-19 patients, and 181 COVID-19 patients leaving a total of 166 healthy volunteers, 60 non-COVID-19 patients, and 241 COVID-19 patients including 184 mild and moderate patients, and 57 severe and critically severe patients in the study (see Flowchart in Additional file 1: Online Figure 1). The characteristics of the healthy group, non-COVID-19 patients, mild and moderate COVID-19 patients, and severe and critically severe COVID-19 patients are provided in Additional file 1: Online Table 3.

Severe and critically severe COVID-19 patients presented with abnormal platelet parameters, including decreased platelet counts and plateletcrit (PCT), increased mean platelet volume (MPV), and platelet distribution width (PDW) as well as abnormal coagulation parameters including increased prothrombin time (PT), international normalized ratio (INR), activated partial thromboplastin time (APTT), d-dimer and fibrinogen degradation products (FDPs), and decreased prothrombin time activity (PTA) when compared with healthy donors, non-COVID-19 patients, and mild and moderate COVID-19 patients (Additional file 1: Online Table 3). Similar results were observed after propensity score matching of severe and critically severe COVID-19 patients to the other groups (Additional file 1: Online Table 4). In addition, mild and moderate COVID-19 patients presented with decreased platelet counts and increased MPV, compared with healthy donors and non-COVID-19 patients. Similar results were observed after propensity score matching (Additional file 1: Online Table 3 and 5). MPV was shown to correlate with platelet activity and is considered a marker of platelet activity [49, 50]; therefore, increased MPV in COVID-19 suggests that these platelets may present as hyperactive.

**Fig. 1:**
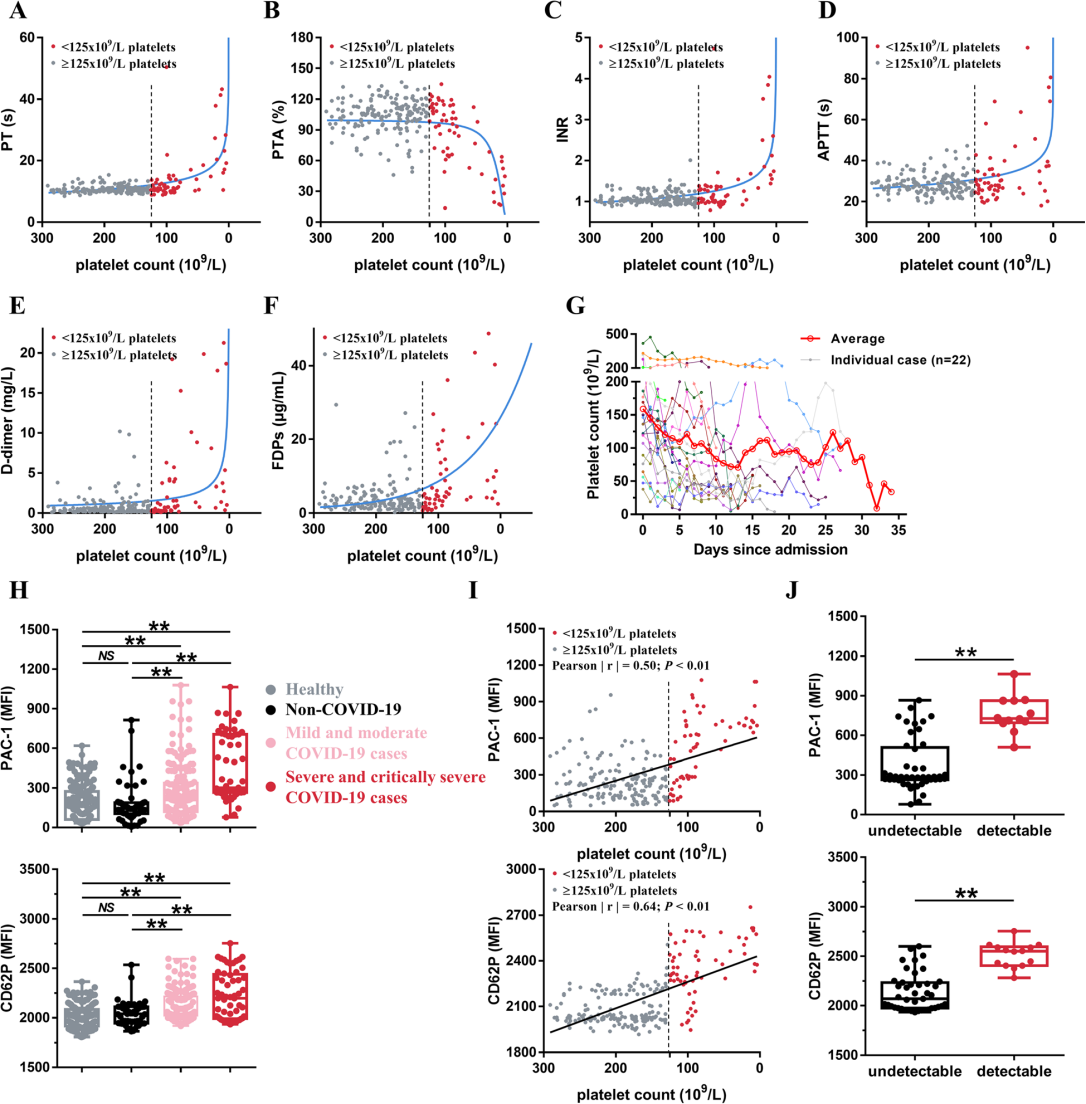
Increased platelet activation in patients with SARS-CoV-2 infection. **a**–**f** Dot plot showing the correlation between platelet count and PT (**a**), platelet count and PTA (**b**), platelet count and INR (**c**), platelet count and APTT (**d**), platelet count and d-dimer (**e**), as well as platelet count and FDPs (**F**) in COVID-19 patients (*n* = 241). Each circle represents a different patient. **g** Dynamics of platelet count in COVID-19 patients with critically severe illness after hospital admission. The platelet counts values were obtained from 22 independent patients. Different colors were used for different patients. **h** Increased expression of platelet integrin αIIbβ3 activation (PAC-1 binding) and P-selectin (CD62P) expression in COVID-19 patients compared with healthy 11 donors and non-COVID-19 patients. Each circle represents a different individual from healthy donors (*n* = 166), non-COVID-19 cases (*n* = 60), the mild and moderate COVID-19 cases (*n* = 184) or the severe and critically severe COVID-19 cases (*n* = 57). **I**, PAC-1 binding and CD62P expression are correlated with platelet count in COVID- 19 patients (*n* = 241). Each solid circle represents a different individual. **j** PAC-1binding and CD62P expression in severe and critically severe type COVID-19 patients with detectable blood virus RNA (detectable, *n* = 12) and with undetectable blood virus RNA (undetectable, *n* = 45). Statistical analyses were performed using Kruskal-Wallis test with Bonferroni correction in (**h**), Pearson’s correlation analysis in (**i**) and nonparametric Mann-Whitney *U* test in (**j**). *NS* no significance; **P < 0.01. *PT* prothrombin time, *PTA* prothrombin time activity, *INR* international normalized ratio, *APTT* activated partial thromboplastin time, *FDPs* fibrinogen degradation products, undetectable: severe and critically severe type COVID-19 patients with undetectable blood virus RNA, detectable: severe and critically severe type COVID-19 patients with detectable blood virus RNA

The relationship between the platelet count and coagulation parameters in COVID-19 patients is described in Fig. 1a–f. For those with a normal platelet count (> 125 × 10^9^/L), the platelet count did not significantly impact the outcomes of the PT, PTA, INR, APTT, d-dimer, and FDPs tests. However, in patients with thrombocytopenia (< 125 × 10^9^/L), when the platelet count decreases, PT, INR, APTT, d-dimer, and FDPs increase exponentially, while PTA decreases exponentially. We further examined platelet count over time in severe and critically severe COVID-19 patients (*n* = 22). Our results suggest that platelet count decreases gradually after hospital admission (Fig. 1g).

Consistent with the increased MPV, we found that integrin αIIbβ3 activation (PAC-1 binding) and P-selectin (CD62P) expression were increased in platelets of COVID-19 patients (Fig. 1h, Additional file 1: Online Figure 2). The severe and critically severe COVID-19 patients presented the highest integrin αIIbβ3 activation and P-selectin expression on platelets. Platelet activation leads to platelet consumption which causes thrombocytopenia [51]; notably, PAC-1 binding and CD62P expression were both moderately correlated with decreases in platelet count (Pearson │*r*│ for PAC-1 = 0.50, *P* < 0.01; Pearson │*r*│ for CD62 = 0.64, *P* < 0.01; Fig. 1i) in COVID-19 patients.

### Detectable COVID-19 viral RNA in blood is an indicator of platelet hyperactivity in severe and critically severe COVID-19 patients

Of the 241 COVID-19 patients, a total of 15 (6.22%) patients, including 12 severe and critically severe and 3 mild and moderate cases of COVID-19, were positive for blood SARS-CoV-2 RNA. In severe and critically severe patients, patients with detectable blood viral RNA presented with higher integrin αIIbβ3 activation and P-selectin expression on platelets, compared with those with undetectable blood viral RNA (Fig. 1j). To test the effect of SARS-COV-2 RNA positive blood on platelet aggregation, we incubated healthy platelets with SARS-COV-2 RNA-positive platelet-poor plasma (PPP) and SARS-COV-2 RNA-negative PPP from severe and critically severe patients, and used healthy PPP as control. We found that SARS-COV-2 RNA-positive PPP enhanced platelet aggregation, compared with SARS-COV-2 RNA-negative PPP and healthy PPP (Additional file 1: Online Figure 3). These results suggest that the presence of SARS-CoV-2 viral RNA in blood is an indicator of platelet hyperactivity.

### Human and mouse platelets express ACE2 and TMPRSS2

**Fig. 2:**
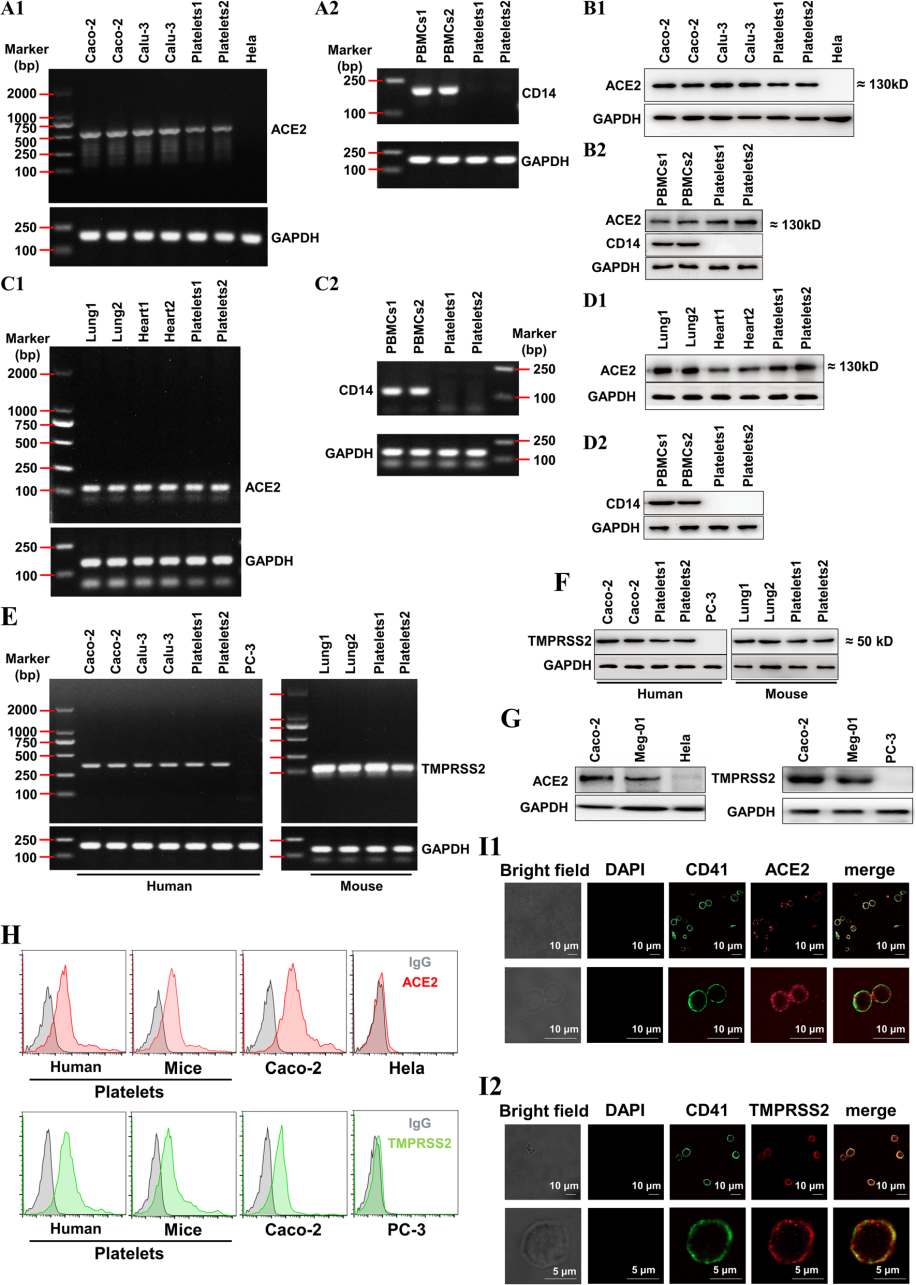
Both human 1 and mouse platelets express ACE2 and TMPRSS2. A, RT2 PCR detection of ACE2 (A1) and monocyte-specific CD14 (A2) in healthy human platelets. B, Western blot detection of ACE2 and monocyte-specific CD14 (B2) in healthy human platelets. For A and B, the human colon cell line Caco-2 and the human lung cell line Calu-3 were used as positive controls of ACE2, and the human Hela cell line was used as a negative control of ACE2. The peripheral blood mononuclear cells (PBMCs) from healthy human were used as a positive control of CD14. C, RT-PCR detection of ACE2 (C1) in lungs, hearts, and platelets from wild-type mice. D, Western blot detection of ACE2 in lungs, hearts, and platelets from wild-type mice. For C and D, PBMCs from mice were used as a positive control of CD14. E, RT-PCR detection of TMPRSS2 in platelets from healthy human and wild-type mice. F, Western blot detection of TMPRSS2 in platelets from healthy human and wild-type mice. For E and F, the colon cell line Caco-2 and the human lung cell line Calu-3 from human and the lungs from mice were used as positive controls of TMPRSS2, and the human prostate cell line PC-3 was used as a negative control of TMPRSS2. For A to F, platelet-rich plasma prepared as previously described was filtered through a Sepharose 2B column equilibrated in Tyrode’s solution to isolate platelets. Platelets1 in A, B, E left panel and F left panel were platelets from 1 healthy blood sample and platelets2 in A, B, E left panel and F left panel were platelets mixture from 20 healthy donors. Platelets1 in C, D, E right panel and F right panel were platelets from 1 wild-type mouse and platelets2 in C, D, E right panel and F right panel were platelets mixture from 5 wild-type mice. PBMCs were isolated by centrifugation on a Ficoll-Paque from two different blood samples of healthy donors (PBMCs1 and PBMCs2 in A and B) and from two different blood samples of wild-type mice (PBMCs1 and PBMCs2 in C and D). The two different lung (lung1 and lung2 in C, D, E and F) and heart (heart1 and heart2 in C and D) tissues were dissected from different wild-type mice. G, Western blot detection of ACE2 and TMPRSS2 in megakaryocyte cell line (Meg-01). H, Detecting ACE2 and TMPRSS2 expression on healthy human and wild-type mice platelets by flow cytometry. I, Imaging of ACE2 (I1) and TMPRSS2 (I2) expression in healthy human platelets using confocal microscopy. ACE2, the angiotensin converting enzyme 2; TMPRSS2, transmembrane protease serine 2; and RT-PCR, reverse transcription polymerase chain reaction. Images were representative of three independent RT-PCR, Western blot or flow cytometry experiments

Since SARS-CoV-2 infects host cells via ACE2, we explored whether platelets express ACE2. We found that human platelets exhibit robust expression of ACE2 at both the RNA and protein levels as detected by RT-PCR (Fig. 2(A1)) and Western blot (Fig. 2(B1)). These levels were similar to the human colon cell line Caco-2 and the human lung cell line Calu-3, which are used as SARS-CoV-2-infected host cells [52, 53]. The HeLa cell line was used as a negative control for ACE2 expression [54]. The detected ACE2 in platelets is not from the contaminated PBMCs, because the PBMCs-specific marker CD14 was not detectable in our platelet samples when evaluated by RT-PCR (Fig. 2(A2)) or Western blot (Fig. 2(B2)). ACE2 level in platelets is comparable with that in PBMCs (Fig. 2(B2)). Mouse platelets exhibited abundant expression of ACE2, similar to lung and heart tissues at the RNA (Fig. 2(C)) and protein levels (Fig. 2(D)).

A recent study reported that cellular serine protease TMPRSS2 (transmembrane protease serine 2) primes SARS-CoV-2 for cell entry [53]. Interestingly, we detected robust expression of TMPRSS2 in platelets comparable with that in human Caco-2 cells, human Calu-3 cells, and mouse lung tissues at the RNA (Fig. 2(E)) and protein levels (Fig. 2(F)). Furthermore, abundant ACE2 and TMPRSS2 protein expression was confirmed in platelet progenitor megakaryocyte cells (Meg-01 cell line, Fig. 2(G)). Finally, ACE2 and TMPRSS2 protein expression was further confirmed in human and mouse platelets using flow cytometry (Fig. 2(H)) and in human platelets using confocal immunofluorescence (Fig. 2(I)).

### SARS-CoV-2 virus directly potentiates platelet activation

**Fig. 3:**
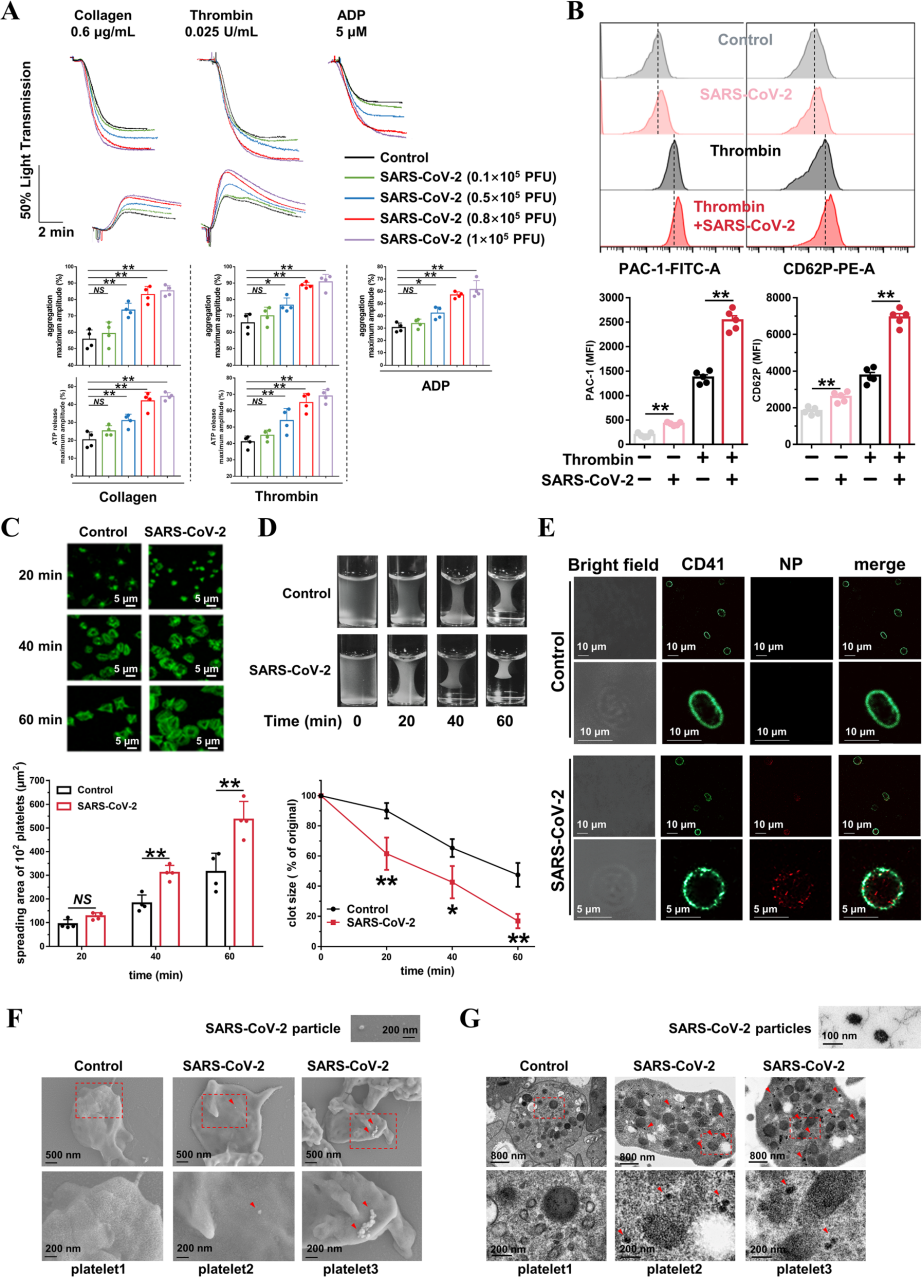
SARS-CoV-2 directly enhances 1 platelet activation in vitro. **a** SARS-CoV-2 dose-dependently potentiated platelets aggregation and ATP release in response to collagen, thrombin, and ADP in vitro. Washed platelets from healthy donors were preincubated with SARS-CoV-2 in the indicated concentration for 30 min, then stimulated with collagen (0.6 μg/mL), thrombin (0.025 U/mL), or ADP (5 μM). Aggregation and ATP release (with luciferase) were assessed under stirring at 1200 rpm. Representative results and summary data of 4 experiments are presented. **b** SARS8 CoV-2 induced PAC-1 binding and CD62P expression in the absence of agonist; and potentiated integrin PAC-1 binding and CD62P expression induced by thrombin in platelets. Platelets were preincubated with SARS-CoV-2 virus (1 × 10^5^ PFU, 60 min) or with SARS-CoV-2 virus (1×10^5^ PFU, 30 min), and treated with thrombin (0.025 U/mL, 10 min), and then analyzed using a flow cytometer. Representative flow cytometry histograms and summary data of 5 experiments are presented. **c** Representative confocal fluorescence images (phalloidin) showing that SARS-CoV-2 potentiated platelet spreading on immobilized fibrinogen (100 μg/mL). After preincubation with SARS-CoV-2 (1 × 10^5^ PFU) for 30 min, platelets were allowed to spread on the fibrinogen-coated surfaces at 37 °C for indicated times. Representative results and summary data of 4 experiments are presented. **d** SARS-CoV-2 potentiated clot retraction induced by thrombin. Platelets from healthy donors were normalized at a concentration of 4 × 10^8^/mL and preincubated with SARS-CoV-2 (1 × 10^5^ PFU) for 30 min, then stimulated with thrombin (1 U/mL). Representative results and summary data of 4 experiments are presented. **e** Immunofluorescent staining of Nucleocapsid protein (NP, red) and CD41 (green) in human platelets incubated with SARS-CoV-2 virus (1 × 10^5^ PFU) for 3 h. Representative images from 3 experiments using platelets from different healthy donors. **f** Scanning electron microscope (SEM) of SARS-CoV-2 particles on the surface of platelets. SEM of healthy human platelets (3 × 10^8^ platelets/mL) incubated with SARS-CoV-2 (1 × 10^5^ PFU) for 30 min. Platelets were washed for 3 times and fixed immediately after incubation and processed for SEM experiment. Representative images of single platelet from control group (platelet1) and SARS-CoV-2 treatment group (platelet2 and platelet3) are shown from three different experiments. Arrows point toward the SARS-CoV-2 virus. **g**. Transmission electron microscopy (TEM) of SARS-CoV-2 particles in platelets. TEM of healthy human platelets (3 × 10^8^ platelets/mL) incubated with SARS-CoV-2 (1 × 10^5^ PFU) for 3 h. Platelets were washed for 3 times and fixed immediately after incubation and processed for TEM experiment. Representative images from control group (platelet1) and SARS CoV-2 treatment group (platelet2 and platelet3) are shown from three different experiments. Arrows point toward the SARS-CoV-2 particles. Statistical analyses were performed using unpaired two-tailed Student’s t test in (**a**), (**b**) and (**c**). *NS* no significance; *P < 0.05; **P < 0.01. Two-way ANOVA and Tukey’s post hoc test was performed in (**d**); *P < 0.05 and **P < 0.01 compared with control group

After providing evidence that platelets express ACE2 and TMPRSS2, we went on to evaluate whether SARS-CoV-2 virus could directly activate platelets. We found that incubation with SARS-CoV-2 (1 × 10^5^ PFU [36]) for 30 min did not induce platelet aggregation in human washed platelets from healthy donors (Additional file 1: Online Fig. 4), but in the range of 0.1 to 1 × 10^5^ PFU, SARS-CoV-2 dose-dependently potentiated platelet aggregation in response to collagen (0.6 μg/mL), thrombin (0.025 U/mL), and ADP (5 μmol/L). Consistently, SARS-CoV-2 increased platelet dense granule secretion (ATP release) in response to collagen and thrombin in a dose-dependent manner (Fig. 3a). SARS-CoV-2 induced integrin αIIbβ3 activation and P-selectin expression under basal conditions and enhanced both integrin αIIbβ3 activation and P-selectin expression following agonist activation (Fig. 3b).

We then went on to assess platelet spreading on immobilized fibrinogen and clot retraction and found that pre-incubation with SARS-CoV-2 markedly enhanced platelet spreading (Fig. 3c) and clot retraction (Fig. 3d). In addition, we observed SARS-CoV-2 particles attached to platelet membrane using SEM (Fig. 3f), indicating that SARS-CoV-2 can bind directly to platelets. Moreover, fluorescent confocal microscopy and TEM revealed that SARS-CoV-2 particles were present inside the platelets (Fig. 3e, g), suggesting that SARS-CoV-2 can infect these cells.

### SARS-CoV-2 virus directly induces decrease in platelet ACE2

ACE2 degradation plays an important role in the pathogenesis of COVID-19. SARS-CoV2 has been reported to promote ACE2 internalization and subsequent degradation [55]. Similarly, we found that SARS-CoV-2 induced a time-dependent decrease in ACE2 levels in platelets (Additional file 1: Online Figure 5a), indicating the degradation of ACE2 in platelets upon ACE2 activation. Consistently, we found that ACE2 levels were decreased in platelets from COVID-19 patients, compared with healthy donors. Severe and critically severe COVID-19 patients presented the lowest level of ACE2 expression (Additional file 1: Online Figure 5b).

### SARS-CoV-2 Spike protein directly potentiates platelet activation

**Fig. 4:**
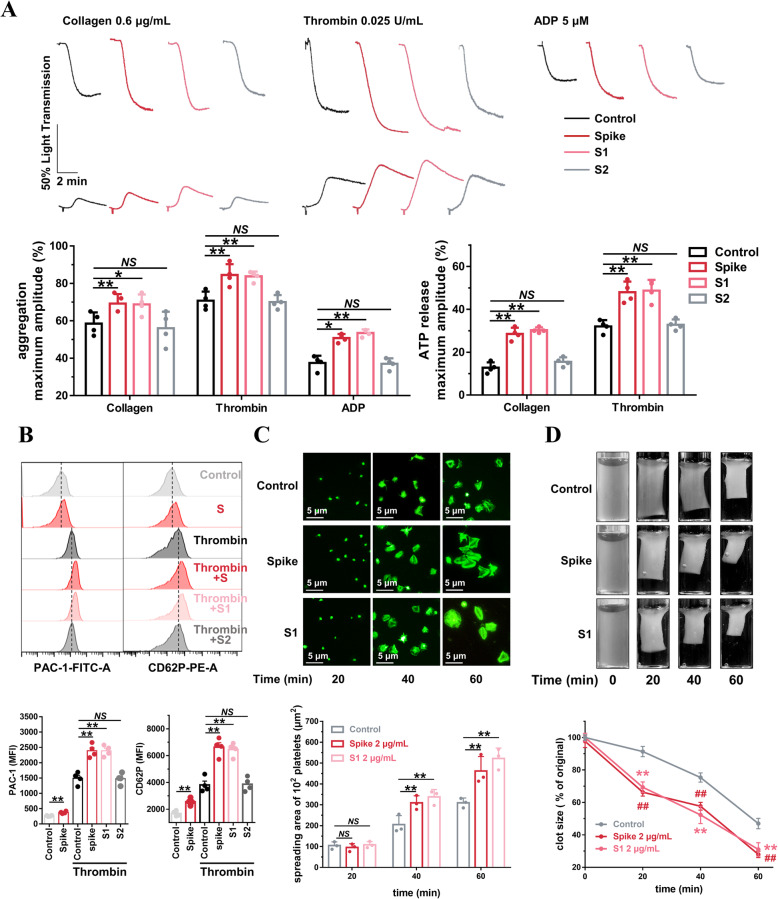
SARS-CoV-2 Spike 1 protein directly enhances human platelet activation. **a** SARS-CoV-2 Spike protein and Spike subunit 1 (S1) potentiated platelet aggregation and ATP release in response to collagen, thrombin, and ADP in vitro, whereas Spike subunit 2 (S2) did not. Washed platelets from healthy donors were preincubated with Spike protein, S1 or S2 at 2 μg/mL for 5 min, then stimulated with collagen (0.6 μg/mL), thrombin (0.025 U/mL), or ADP (5 μM). Aggregation and ATP release (with luciferase) were assessed under stirring at 1200 rpm. Representative results and summary data of 4 experiments are presented. **b** Spike protein stimulated platelets for PAC-1 binding and CD62P expression in the absence of agonist. In addition, Spike protein and S1 increased PAC-1 binding and CD62P expression induced by thrombin in platelets. Platelets were incubated with Spike protein (2 μg/mL, 60 min) in the absence of agonist, or preincubated with Spike protein, S1 or S2 at 2 μg/mL for 5 min and stimulated with thrombin (0.025 U/mL) for 10 min, and then analyzed using a flow cytometer. Representative results and summary data of 4 experiments are presented. **c** Spike protein (2 μg/mL) and S1 (2 μg/mL) potentiated platelet spreading on immobilized fibrinogen. After preincubation with Spike protein (2 μg/mL) for 5 min, platelets were allowed to spread on the fibrinogen-coated surfaces at 37 °C for the indicated times. Representative results and summary data of 3 experiments are presented. **d** Spike protein and S1 potentiated clot retraction induced by thrombin. Platelets from healthy donors were normalized at a concentration of 4 × 10^8^/mL and preincubated with Spike protein (2 μg/mL) or S1 (2 μg/mL) for 5 min, then stimulated with thrombin (1 U/mL). Representative images and summary data are presented from 3 experiments using platelets from different donors. Statistical analyses were performed using one-way ANOVA, followed by Tukey’s post hoc analysis in (**a**), (**b**) and (**c**). *NS* no significance; *P < 0.05; **P < 0.01. Two-way ANOVA and Tukey’s post hoc test was performed in (**d**); ## significant difference (*P* < 0.01) between Spike and control group; ** significant difference (*P* < 0.01) between S1 and control group

Since SARS-CoV-2 binds to host cells via interactions between the Spike protein and ACE2, we sought to explore whether Spike protein could regulate platelet function. Incubation with Spike protein for 9 min did not induce platelet aggregation in human washed platelets from healthy donors (Additional file 1: Online Figure 4). However, similar to the results from the SARS-CoV-2 virus experiments, we were able to demonstrate that the Spike protein dose-dependently enhanced platelet aggregation and ATP release (Additional file 1: Online Figure 6). We further found that SARS-CoV-2 Spike protein (2 μg/mL, 5 min), Spike protein subunit 1 (S1, 2 μg/mL, 5 min), but not Spike protein subunit 2 (S2, 2 μg/mL, 5 min), potentiated platelet aggregation and dense granule secretion in response to different agonists (Fig. 4a). Using flow cytometry, we found that Spike protein induced integrin αIIbβ3 activation and P-selectin expression in the absence of agonist. Furthermore, Spike protein and S1, but not S2, enhanced both integrin αIIbβ3 activation and P-selectin expression in the presence of agonist (Fig. 4b). These data indicate that S1, but not S2, binds ACE2 to regulate platelet function, which corroborates the finding that the receptor-binding domain (RBD) of the Spike protein is found in the S1 subunit [56]. After incubation with Spike or S1 protein, platelets also displayed markedly accelerated spreading (Fig. 4c) and clot retraction (Fig. 4d).

### SARS-CoV-2 directly activates the ACE2/mitogen-activated protein kinase pathway to potentiate platelet activation

**Fig. 5:**
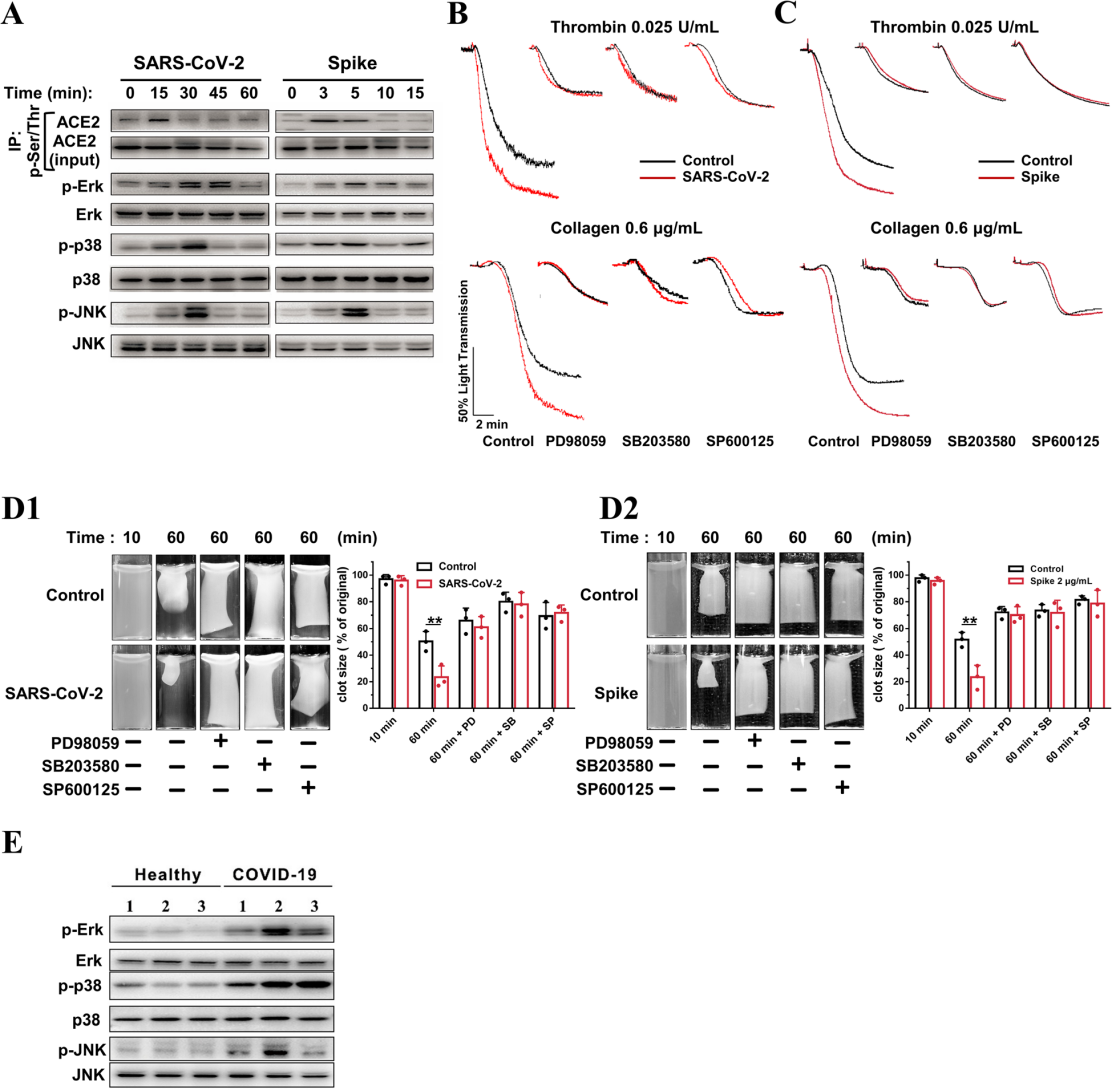
ACE2/MAPK mediates the 1 potentiating effects of SARS-CoV-2 on platelet activation. A SARS-CoV-2 virus and its Spike protein phosphorylate ACE2, Erk, p38, and JNK in human platelets. Platelets from healthy donors were pretreated with SARS-CoV-2 (1 × 10^5^ PFU) or its Spike protein (2 μg/mL) for various times, as 5 indicated. For ACE2 phosphorylation detection, cell lysates were prepared and subjected to immunoprecipitation (IP) with anti-phospho-Ser/Thr antibody, followed by immunoblotting analysis with anti-ACE2 antibody. Cell lysates without the process of immunoprecipitation (10% of input) were analyzed in parallel as loading controls. For p-Erk, p-p38, and p-JNK detection, cell lysates were prepared and directly subjected to immunoblotting. Representative results are presented from 4 experiments using platelets from different healthy donors and summary data is presented in the Additional file 1: Online Figure 7. B Enhanced platelet aggregation by SARS-CoV- 2 is abolished by MAPK inhibitors. The healthy human platelets were pretreated with 10 μM PD98059 (ERK1/2 inhibitor), 10 μM SB203580 (p38 inhibitor), or 10 μM SP600125 (JNK inhibitor) for 10 min, then treated with SARS-CoV-2 (1 × 10^5^ PFU, 30 min) before stimulation by 0.025 U/mL thrombin or 0.6 μg/mL collagen. Representative results are presented from 3 experiments using platelets from different donors. C Enhanced platelet aggregation by Spike protein is abolished by MAPK inhibitors. Human platelets were pretreated with 10 μM PD98059, 10 μM SB203580, or 10 μM SP600125 for 10 min, then treated with Spike protein (2 μg/mL, 5 min) or vehicle as control before stimulation by 0.025 U/mL thrombin or 0.6 μg/mL collagen. Representative results are presented from 3 experiments using platelets from different healthy donors. D Accelerated clot retraction by SARS-CoV-2 (D1) or its Spike protein (D2) is abolished by MAPK inhibitors. Platelets were pretreated with 10 μM PD98059, 10 μM SB203580, or 10 μM SP600125 for 10 min, and then incubated with SARS26 CoV-2 (1 × 10^5^ PFU, 30 min) or Spike protein (2 μg/mL, 5 min) as in B and C. Clot retraction was initiated as in Fig. 3d. Representative images and summary data of 3 experiments are presented using platelets from different healthy donors. Representative images and summary data are presented from 3 experiments using platelets from different donors. E Increased phosphorylation of Erk, p38, and JNK in platelets from COVID-19 patients, compared with healthy donors. Representative results are presented using platelets from 6 individuals from different COVID-19 patients (*n* = 3) and healthy donors (*n* = 3). Statistical analyses were performed using unpaired two34 tailed Student’s *t* test in (D1) and (D2). *P < 0.05; **P < 0.01. MAPK indicates mitogen activated protein kinase; PD, PD98059; SB, SB203580; SP, SP600125

Mitogen-activated protein kinase (MAPK) has been well documented in platelet activation and thrombosis. ACE2/MAPK pathway activation has been reported to mediate SARS-CoV-2-induced cytokine modulation in lung cells [27, 57]. Thus, we examined whether the ACE2/MAPK pathway is involved in SARS-CoV-2-mediated platelet activation. SARS-CoV-2 and Spike protein stimulated platelet ACE2 phosphorylation at 15 and 3 min, respectively (Fig. 5(A) and Additional file 1: Online Figure 7a). MAPK (Erk, p38, and JNK) kinase was phosphorylated apparently later than ACE2 phosphorylation (Fig. 5(A) and Additional file 1: Online Figure 7b). These results agree with a previous study conducted on lung cells [27] and supports the hypothesis that the MAPK pathway is activated downstream of ACE2 in platelets.

We investigated whether MAPK signaling mediates the potentiating effects of SARS-CoV-2 on platelet activation. As shown in Fig. 5(B and, C), the inhibition of Erk, p38, and JNK with PD98059 (10 μM), SB203580 (10 μM), and SP600125 (10 μM) abolished the potentiating effects of SARS-CoV-2 (Fig. 5(B)) or Spike protein (Fig. 5(C)) on agonist-induced platelet aggregation. In addition, we observed that the accelerated clot retraction elicited by SARS-CoV-2 or Spike protein was also prevented by Erk, p38, or JNK inhibitors (Fig. 5(D)).

We further detected MAPK phosphorylation in platelets from COVID-19 patients. Our results demonstrated that COVID-19 patients presented increased phosphorylation of Erk, p38, and JNK in platelets, compared with healthy donors (Fig. 5).

Together, these data suggest that MAPK is phosphorylated downstream of ACE2 and may mediate the potentiating effects of SARS-CoV-2 on platelet activation.

### SARS-CoV-2 Spike protein enhances thrombosis potential in vivo

**Fig. 6:**
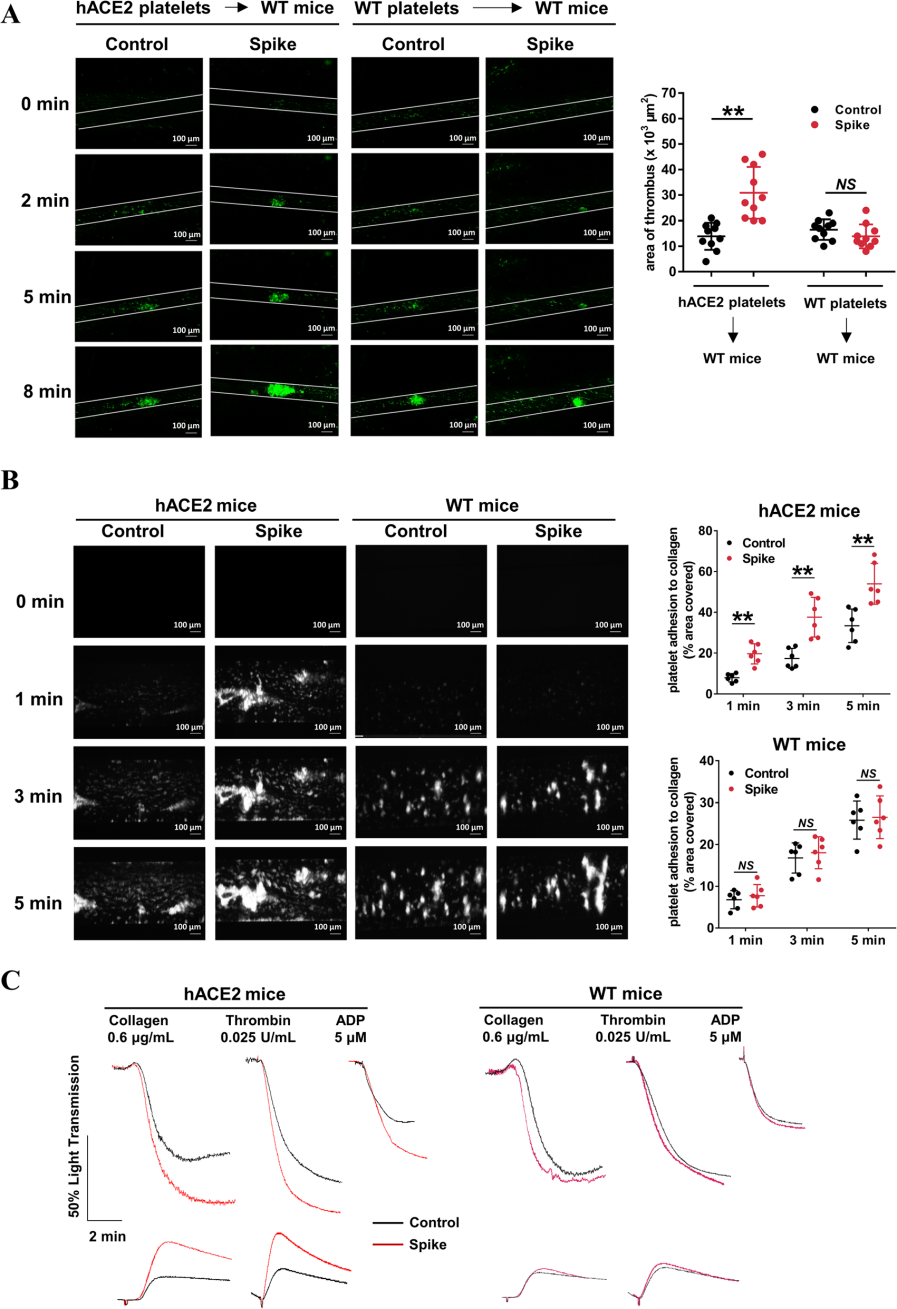
SARS-CoV-2 Spike 1 protein directly enhances thrombosis potential in vivo. **a** Washed platelets from wild-type or hACE2 transgenic mice were infused into WT mice. After intravenous injection 200 μg/kg Spike protein or control (saline), FeCl3-induced arterial thrombus formation was initiated, and the thrombus area was recorded. Representative image of thrombus formation and the relative fluorescence at different time points are shown. Statistically analysis of FeCl3-induced thrombosis by assess thrombus area at 8 min (*n* = 10). **b** Spike protein-treated whole blood from hACE2 mice showed accelerated thrombus formation over an immobilized collagen surface at a shear rate of 1000 s^−1^, whereas Spike protein-treated whole blood from wild type mice did not. The whole blood from mice was fluorescently labeled by mepacrine (100 μM, 30 min) and incubated with Spike protein (2 μg/mL) for 5 min, and then perfused through fibrillar collagen-coated bioflux plates for 5 min. Representative images and time courses of thrombus formation challenged with control or Spike protein at the indicated time points are presented. Dot plot showing thrombus formation area (*n* = 6). **c** Spike protein-treated platelets from hACE2 mice presented increased platelet aggregation and ATP release in response to collagen, thrombin, and ADP, whereas Spike protein-treated platelets from wild-type mice did not. Washed platelets from mice were pretreated with control or Spike protein (2 μg/mL) for 5 min, and then stimulated with collagen (0.6 μg/mL), thrombin (0.025 U/mL), or ADP (5 μM). Aggregation and ATP release (with luciferase) were assessed under stirring at 1200 rpm. Representative results are presented from 4 experiments using platelets from different mice and summary data are presented in Additional file 1: Online Figure 8. Statistical analyses were performed using unpaired two-tailed Student’s *t* test in (**a**) and (**b**). *NS* no significance; **P < 0.01. WT indicates wild-type; hACE2 indicates hACE2 transgenic

We then tested whether SARS-COV-2 Spike protein activates platelets and thereby enhances thrombus formation in vivo. To eliminate non-platelet hemostatic or thrombotic factor variations, we injected 10^9^ platelets from transgenic mice bearing human ACE2 (hACE2) or wild-type mice into thrombocytopenic wild-type mice. We simultaneously intravenously injected 200 μg/kg SARS-CoV-2 Spike protein into mice [30], and examined its effects on FeCl_3_-induced thrombus formation in the mesenteric arterioles. Representative images of the thrombus development at various time points following FeCl_3_ injury are shown in Fig. 6a. Notably, Spike protein had no effect on thrombus formation following mesenteric arteriole injury in mice transfused with platelets from wild-type mice, which agreed with the results that SAR-CoV-2 Spike protein cannot bind mouse ACE2 protein [53]. However, Spike protein did potentiate thrombus formation in mice transfused with platelets from hACE2 transgenic mice.

We then assessed the role of the Spike protein on mice platelets for thrombus formation under arterial flow conditions using a microfluidic whole-blood perfusion assay. Throughout the perfusion period, thrombus formation was significantly increased when pre-treated with Spike protein (2 μg/mL, 5 min), in whole blood from hACE2 transgenic mice, but not in whole blood from wild-type mice (Fig. 6b). In addition, the Spike protein potentiated platelet aggregation and ATP release in response to agonists in vitro and enhanced thrombosis formation in vivo on hACE2 transgenic mice, while it had no effect on wild-type mice (Fig. 6c and Additional file 1: Online Figure 8).

### SARS-CoV-2 directly induces the release of coagulation factors, inflammatory cytokines, and the formation of leukocyte-platelet aggregates

Platelets are an important source of coagulation factors V and XIII in α granules, and serve as adhesion sites for coagulation factors activation via their surface exposure of phosphatidylserine (PS) [58]. We demonstrated that SARS-CoV-2 stimulated platelets to release both Factor V (Additional file 1: Online Figure 9A) and Factor XIII (Additional file 1: Online Figure 9B), at levels that were comparable to thrombin stimulation. However, SARS-CoV-2 had no effect on platelet PS exposure (Additional file 1: Online Figure 9C). Similar results were also observed in the Spike protein-treated platelets (Additional file 1: Online Figure 9D, 9E, and 9F).

Platelets carry a large variety of inflammatory cytokines in α granules, which can be quickly secreted upon activation to participate in the immune response [59]. We found that SARS-CoV-2 and its Spike protein stimulated PF4 (Additional file 1: Online Figure 10A), TNF-α (Additional file 1: Online Figure 10B), IL-8 (Additional file 1: Online Figure 10C), and IL-1β (Additional file 1: Online Figure 10D) secretion from platelets. Consistently, we found that plasma PF4 levels were increased in COVID-19 patients, compared with healthy donors. The severe and critically severe COVID-19 patients presented the highest plasma PF4 levels (Additional file 1: Online Figure 10E).

P-selectin is a key adhesion molecule that mediates the interaction between platelets and leukocytes via P-selectin glycoprotein ligand 1 (PSGL-1). Consistent with our finding that SARS-CoV-2 increased P-selectin expression, we found that SARS-CoV-2 and Spike protein increased the proportion of leukocyte–platelet aggregates (LPAs) (CD45^+^CD41^+^ aggregates, Additional file 1: Online Figure 11). Specifically, both monocyte–platelet aggregates (MPAs, CD14^+^CD41^+^) and neutrophil-platelet aggregates (NPAs, CD65^+^CD41^+^) were significantly increased after SARS-CoV-2 or Spike protein treatment.

### Recombinant human ACE-2 protein and an anti-Spike monoclonal antibody suppress SARS-CoV-2-induced platelet activation

**Fig. 7:**
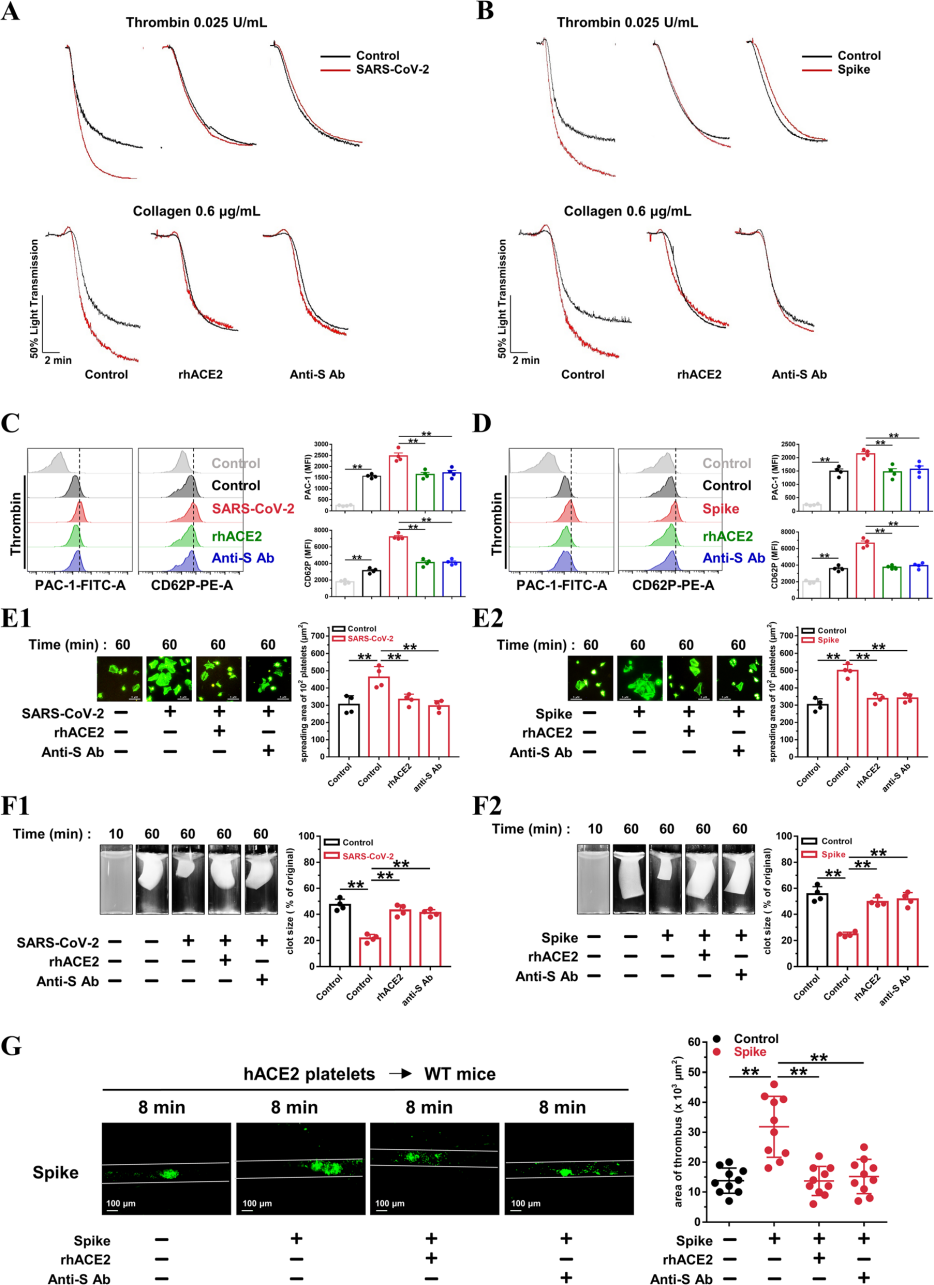
Recombinant human ACE2 protein and anti-Spike monoclonal antibody suppress SARS-CoV-2-induced platelet activation. A, Enhanced platelet aggregation by SARS-CoV-2 is abolished by recombinant human ACE2 protein and anti-Spike monoclonal antibody (targeting the receptor-binding domain [RBD] of SARS-CoV-2). Representative results are presented from 3 experiments using platelets from different donors. B, Enhanced platelet aggregation by Spike protein is suppressed by ACE2 protein and anti-Spike antibody. Representative results are presented from 3 experiments using platelets from different healthy donors. For A and B, the healthy human platelets were pretreated with ACE2 protein (10 μg/mL) or anti-Spike antibody (4 μg/mL) for 10 min, then treated with SARS-CoV-2 (1 × 10^5^ PFU, 30 min) or Spike protein (2 μg/mL, 5 min) before stimulation by 0.025 U/mL thrombin or 0.6 μg/mL collagen. C and D, The ACE2 protein and anti-Spike antibody reversed PAC-1 binding and CD62P expression induced by SARS-CoV-2 (C) or Spike protein (D). Representative images and summary data are presented from 4 experiments using platelets from different healthy donors. E, Enhanced platelet spreading induced by SARS-CoV-2 (E1) or its Spike protein (E2) are abolished by ACE2 protein and anti-Spike monoclonal antibody. Representative images and summary data of 4 experiments are presented using platelets from different healthy donors. F, Accelerated clot retraction induced by SARS-CoV-2 (F1) or its Spike protein (F2) are abolished by ACE2 protein and anti-Spike monoclonal antibody. Representative images and summary data of 4 experiments are presented using platelets from different healthy donors. For C, D, E and F, platelets were pretreated with ACE2 protein (10 μg/mL) or anti-Spike antibody (4 μg/mL) for 10 min, and incubated with SARS-CoV-2 (1 × 10^5^ PFU, 30 min) or Spike protein (2 μg/mL, 5 min), and then subjected to flow cytometry of thrombin-activated platelets, platelet spreading assay, and clot retraction assay. G, Increased thrombus area induced by Spike protein in wild-type mice transfused with platelets from hACE2 transgenic mice is suppressed by ACE2 protein and anti-Spike antibody. Representative photographs of FeCl3-induced thrombus formation at the indicated time points within 30 min after intravenous administration of Spike protein (200 μg/kg) with ACE2 protein (1 mg/kg) or anti-Spike monoclonal antibody (400 μg/kg). Dot plot showing thrombus area for control or Spike protein treated mice (*n* = 10). Statistical analyses were performed using one-way ANOVA, followed by Tukey’s post hoc analysis in (C), (D), (E), (F), and (G). *P < 0.05; **P < 0.01. RhACE2 indicates recombinant human ACE2 protein; anti-S Ab, anti-Spike antibody

Recombinant human ACE2 protein has been reported to inhibit SARS-CoV-2 infection in engineered human tissues [60]. SARS-CoV-2 neutralizing antibodies produced by the host immune response are a critical part of the toolbox of therapies for COVID-19 [61]. We pretreated platelets from healthy donors with ACE2 protein or an anti-Spike antibody. Both the ACE2 protein and the anti-Spike antibody suppressed SARS-CoV-2 (Fig. 7(A)) or Spike protein (Fig. 7(B))-potentiated platelet aggregation and reversed SARS-CoV-2 or Spike protein-induced PAC-1 binding and CD62P expression (Fig. 7(C and D)). The accelerated platelet spreading (Fig. 7(E)) and clot retraction (Fig. 7(F)) elicited by exposure to SARS-CoV-2 or Spike protein were also prevented by pretreatment with the ACE2 protein and anti-Spike antibody. Of note, treatment with the ACE2 protein and anti-Spike antibody also suppressed the thrombus formation induced by the Spike protein following mesenteric arteriole injury (Fig. 7(G)). These data clearly show that SARS-CoV-2 stimulates platelet activation via the interaction of its Spike protein and ACE2, which can be suppressed by exogenous addition of ACE2 or an anti-Spike antibody.

## Discussion

**Fig. 8:**
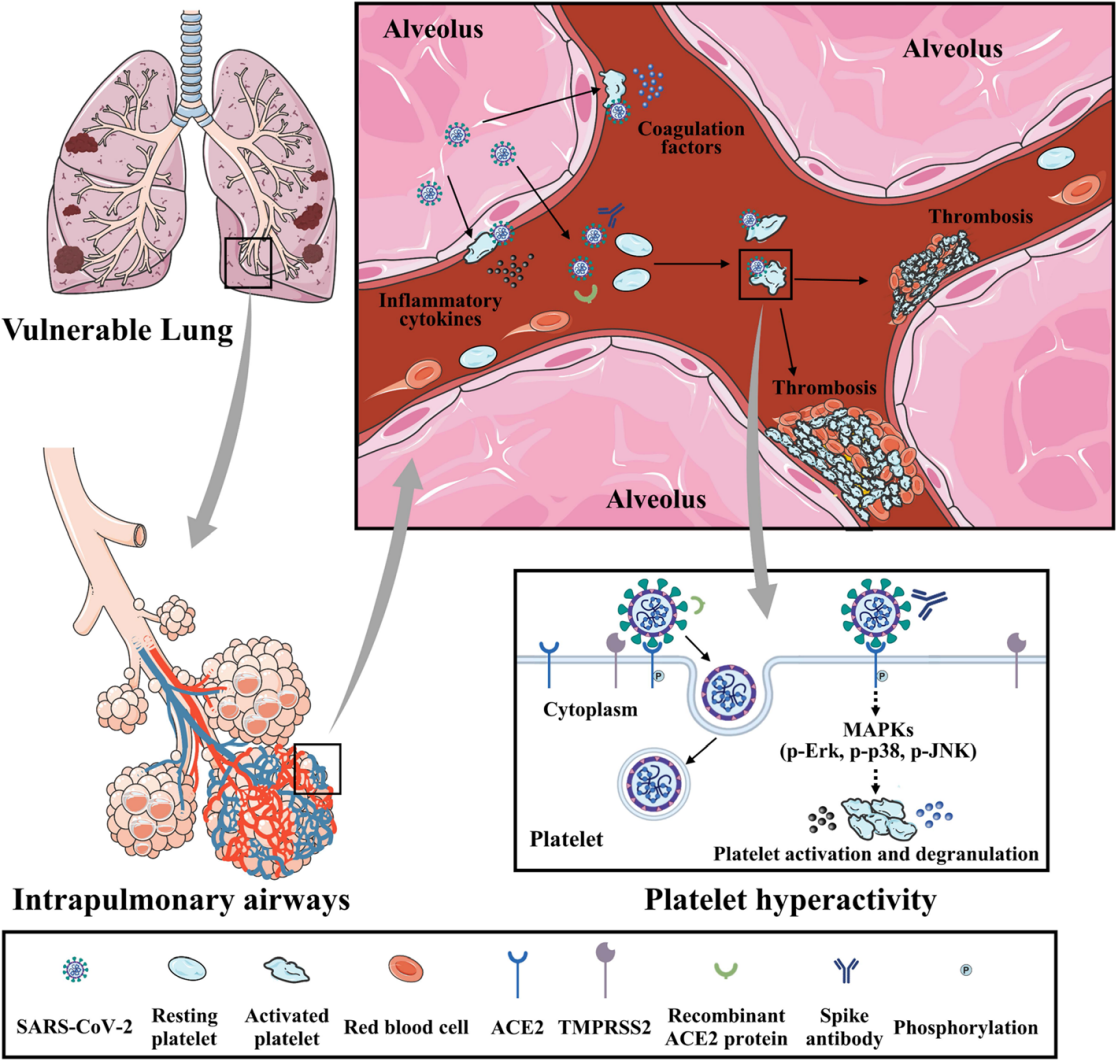
Summary schemes illustrating SARS-CoV-2 activates platelets and enhances thrombosis in COVID-19. Global schema illustrating SARS-CoV-2 from alveolus binds and activates platelets, which enhances thrombosis formation and inflammatory reaction in capillaries, and subsequently contributes to development of disseminated intravascular coagulation and acute respiratory distress syndrome. SARS-CoV-2 Spike protein binds to ACE2 and phosphorylates ACE2, leading to MAPK signaling activation (phosphorylation of Erk, p-38, and JNK) and subsequent platelet activation, coagulation factors release, and inflammatory cytokines secretion. Interaction between SARS-CoV-2 Spike protein and platelet ACE2 confers the platelet activation, which is suppressed by the recombinant human ACE2 protein and anti-Spike monoclonal antibody (central illustration)

Accumulating evidence indicates that COVID-19 predisposes patients to thromboembolic disorders, but a direct association between SARS-CoV-2 and platelet dysregulation has not been reported. In this study, we demonstrated that (1) COVID-19 patients experience increased in vivo platelet activation, as evidenced by increased αIIbβ3 activation and P-selectin expression, and detectable virus RNA in the blood is associated with platelet hyperactivity; (2) platelets robustly express ACE2 and TMPRSS2; (3) SARS-CoV-2 and its Spike protein promote platelet function and thrombus formation via the MAPK pathway downstream of ACE2; and (4) recombinant human ACE2 protein and anti-Spike monoclonal antibody treatment may block SARS-CoV-2-induced platelet activation and thrombus formation. Collectively, these data suggest that SARS-CoV-2-activated platelets may result in the pro-thrombotic state described in COVID-19 patients. Our findings also suggest that reducing platelet hyperactivity via the addition of ACE2 protein and anti-Spike neutralizing antibodies could be an effective therapeutic strategy to prevent thrombotic events in COVID-19 patients (Fig. 8, central illustration).

Emerging evidence has revealed a high composite incidence of thrombotic events in critically ill COVID-19 patients, including venous and arterial thrombotic events, and thrombocytopenia, all of which have been associated with increased mortality [62–64]. Anticoagulation is associated with a reduced risk of mortality without increased bleeding diathesis among patients hospitalized with COVID-19 [65]. However, the underlying mechanism of thrombus formation in COVID-19 is still unclear. We provide evidence that the platelets, key mediators of thrombosis, are hyperactivated in COVID-19 patients. Other recent studies have reported that various platelet activation events, including aggregation, adhesion, infiltration, and inflammatory response, contribute to lung injury and microvascular thrombosis in SARS-CoV-2-associated pneumonia [66–68]. These results, together with those from our study, draw attention to the role of platelet activation in the pathogenesis of COVID-19.

It has long been accepted that viruses indirectly activate platelets during infection by creating an inflammatory microenvironment and subsequent vascular endothelial dysfunction. However, recent studies have shown that there are some direct interactions between certain viruses and platelets, and that these interactions serve as an important supplement for the above-mentioned activation [69]. Platelets bind to encephalomyocarditis virus via TLR7, to rotavirus via GPIa/IIa, to hantavirus and adenovirus via GPIIb/IIIa, to HIV and DV via lectin receptors such as CLEC-2 and DC-SIGN, and to influenza/IgG immune complexes by FcgRIIA [70]. Here, we provide evidence that platelets express abundant ACE2 and TMPRSS2, two major cellular components responsible for SARS-CoV-2 cell entry.

The recent studies by Manne B K et al. did not detect robust expression of ACE2 in platelets. However, platelets used for the detection of ACE2 in their studies mainly come from COVID-19 patients. Actually, the COVID-19 virus can induce ACE2 internalization and subsequent degradation, evidenced by our study of SARS-CoV-2-induced ACE2 degradation in platelets (Additional file 1: Online Figure 5) and the other studies on SARS-CoV-2-induced ACE2 degradation [30]. Moreover, a recent study indicated that SARS-CoV-2 infection could downregulate ACE2 at the mRNA level and this effect requires action directly on the ACE2 promoter [71]. In consistent with the identification of platelet ACE2 in our results, Zaid et al. also found that ACE2 mRNA was presented in platelets from both healthy people and COVID-19 patients [72].

ACE2 is the primary enzyme responsible for the conversion of Ang II, a pivotal mediator of lung injury, to Ang peptides. SARS-CoV-2 uses ACE2 as its cellular receptor, resulting in ACE2 degradation and ACE/ACE2 imbalance, which could drive Ang II-mediated lung injury in COVID-19 [73, 74]. In addition, the decline of ACE2 in infected cells could confer to a host protective mechanism from further viral attack.

Recent studies reported that platelets are hyperactivated in COVID-19 patients [67, 75, 76]. The platelet activity biomarkers are associated with the coagulation dysfunction [67] and the composite outcome of thrombosis or death [75]. These studies emphasized the notion that cytokine storm may trigger hyperinflammation and hypercoagulability. Previous studies have found that fibrinogen level is higher in COVID-19 patients, which may bind and activate platelets to exacerbate thrombotic disorder in capillaries [77]. Our studies are consistent with the recent finding of platelet hyperactivity in COVID-19 patients. We further extend those finding and suggested a possibility that SARS-CoV-2 virus could directly activate platelets via the interaction of Spike protein and platelet ACE2. We cannot rule out the other possibility that virus-containing immune complex or virus-induced immune mediators may also contribute to platelet hyperactivity in COVID-19, which needs further investigation.

We found that MAPK was phosphorylated in SARS-CoV-2-activated platelets, and ACE2 was phosphorylated earlier than MAPK signaling, indicating that MAPK is activated downstream of ACE2 activation. Supporting our finding, MAPK activation was reported to be attributable to ACE2 signaling in lung cells in an earlier study [27]. We also found that MAPK inhibitors can reverse SARS-CoV-2-induced platelet activation, supporting our hypothesis that MAPK mediates this activation. In line with our findings, recent studies have shown that MAPK phosphorylation promotes thromboxane generation by activating cPLA2 in platelets [78]. MAPK activation also facilitates platelet secretion and clot retraction by stimulating phosphorylation of myosin light chain [79]. We previously reported that MAPK phosphorylation potentiated platelet aggregation and clot retraction [35]. Meanwhile, MAPK inhibition could abolish platelet aggregation induced by a low concentration of agonist [80, 81].

Platelets contain a set of coagulation factors and inflammatory factors, stored in the α-granules that are released upon activation to potentiate the coagulation cascade [82, 83]. We found that SARS-CoV-2 induced CD62P expression, indicating α-granule secretion. In addition, SARS-CoV-2 and its Spike protein directly stimulated Factor V and XIII release as well as LPAs formation. SARS-CoV-2 failed to induce PS exposure, which is consistent with previous reports that suggest that platelet activators are inefficient in inducing PS exposure in the absence of a shear force [84].

## Conclusions

This study showed that ACE2, a host cell receptor for SARS-CoV-2, and TMPRSS2, a serine protease for protein priming, are expressed in platelets, and that the SARS-CoV-2 virus directly activates platelets and potentiates their prothrombotic function and inflammatory response via Spike/ACE2 interactions. Considering the critical roles of platelets in thrombosis, coagulation, and immune response, our study sheds new insight into possible anti-platelet treatment opinions for thrombosis in COVID-19 patients.

### Limitations of study

Our study has several limitations. Firstly, animal experiments of virus invasion are lacked due to the limitations of our experimental conditions. Whether our in vivo thrombus formation study using Spike protein can be also reproduced using living SARS-CoV-2 virus still needs to be further investigated. Secondly, platelets in infected lungs may meet different concentrations of viruses in COVID-19; the in vivo effect of SARS-CoV-2 on platelet activation needs to be further investigated in COVID-19 patients. Thirdly, whether our study in washed platelets can be also reproduced using platelet-rich plasma still needs to be further investigated. Fourthly, the relationship between ACE2 degradation and disease severity, platelet activity, or blood SARS-COV2 RNA existence still needs further investigation.

## Supplementary information

**Additional file 1: **Expanded materials and methods.. Online **Table 1.** Antibodies, staining chemicals and dilutions used in this study. **Online Table 2.** The primers used for RT-PCR**. Online Table 3.** Characteristics of Healthy, Non-COVID-19 patients, Mild and moderate type COVID-19 patients and Severe and critically severe type COVID-19 patients groups before propensity score matching. **Online Table 4.** Patient Characteristics after propensity score matching Sever and critically severe type COVID-19 patients group to healthy group (1:2), Non-COVID-19 patients group (1:1) or Mild and moderate type COVID-19 patients group (1:2). **Online Table 5.** Patient Characteristics after propensity score matching healthy group to Mild and moderate type COVID-19 group (1:1) or Non-COVID-19 patients group to Mild and moderate type COVID-19 group (1:2). **Online Figure 1.** The flowchart showing the strategy of groups enrollment. **Online Figure 2.** Analysis of platelet activation in COVID-19 patients after propensity score matching. A and B, Dot plot showing increased platelet t acti activation (A) and CD62P expression (B) in severe and critically severe COVID-19 patients compared with healthy donors (1:2 matching), non-COVID-19 patients (1:1 matching) or mild and moderate COVID-19 patients (1:2 matching). C and D, Dot plot showing increased PAC-1 binding (C) and CD62P expression (D) in mild and moderate COVID-19 patients compared with healthy donors (1:1 matching), non-COVID-19 patients (2:1 matching). **P < 0.01 vs control. Statistical analyses were performed using Mann-Whitney U test. *P < 0.05; **P < 0.01. **Online Figure 3.** SARS-CoV-2 RNA positive platelet-poor plasma enhanced platelet aggregation compared with SARS-CoV-2 RNA negative platelet-poor plasma and healthy platelet-poor plasma. Healthy platelets were incubated with healthy platelet-poor plasma (PPP), SARS-COV-2 RNA positive PPP and SARS-COV-2 RNA negative PPP from severe and critically severe COVID-19 patients at the concentration of 2oor plasma (PPP), SARS-COV-2 RNA positive PPP and SARS-COV-2 RNA ne experiment. Statistical analyses were performed using one-way ANOVA followed by Tukeyiment. Statistical anal*P* < 0.05, n = 4. **Online Figure 4.** SARS-CoV-2 and its Spike protein did not induce platelet aggregation in the absence of agonist. Platelets were treated with SARS-CoV-2 ((1telets were treated with SARS-CoV-2 not induce plateletway ANOVA followed bygat indicated time in aggregometry. **Online Figure 5.** Platelet ACE2 is reduced upon SARS-CoV-2 treatment. A, Platelets from healthy donors were pretreated with SARS-CoV-2 (1×105 PFU) for various times, as indicated. The ACE2 protein level was detected by Western blot. Representative images and summary data of 3 experiments are presented using platelets from different healthy donors. Statistical analyses were performed using two-way ANOVA and Tukey's post hoc test. **P < 0.01 vs control. B, Decreased ACE2 expression in platelets from COVID-19 patients. Representative images of 12 different individuals from healthy group (n = 4), mild and moderate COVID-19 group (n = 4) and severe and critically severe COVID-19 group (n = 4) are presented. **Online Figure 6.** SARS-CoV-2 Spike protein dose-dependently enhance platelet activation. Washed platelets from healthy donors were incubated with Spike protein in the indicated concentration for 5 min, then stimulated with collagen (0.6 n = 4)e thrombin (0.025 U/mL), or ADP (5 min, then stimulated with collagen (0.6 n = 4)ere were assessed under stirring at 1200 rpm. Representative results and summary data of 4 experiments are presented. Statistical analyses were performed using One-way ANOVA, followed by Tukeyted. Statistical analyses were performed using One-wayata o 0.01. **Online Figure 7.** SARS-CoV-2 and its Spike protein phosphorylates ACE2, Erk, p38, and JNK in human platelets. A, Platelets were challenged with SARS-CoV-2 (1 x 105 PFU) or Spike protein (2 h SARS-CoV-2 (1tes ACE2, Erk,rmed using One-wayata ACE2 normalized to input ACE2, corresponding to Figure 5a, are provided from 4 experiments using platelets from different donors. B, Platelets were challenged with SARS-CoV-2 (1 x 105 PFU) or Spike protein (2 nors. CE2, Erk,rmed using One-wayata p-Erk normalized to Erk, p-p38 normalized to p38, and p-JNK normalized to JNK, corresponding to Figure 5a, are provided from 4 experiments using platelets from different donors. Statistical analyses were performed using One-way ANOVA, followed by Tukeyt donors. Statistical anaA) and (B). *NS*, no significance; *P < 0.05; **P < 0.01. **Online Figure 8.** Spike protein enhances *in vitro* platelet activation and *in vivo* thrombosis potential in hACE2 transgenic mice. A, Quantitative analysis of platelets aggregation and ATP release corresponding to Figure 6C. Data are provided from 4 experiments using platelets from different mice. B, After intravenous injection 200 ter intravenous injectionelets from different mice. ing One-way ANOVA, f formation was initiated, and the thrombus area was recorded. Representative image of thrombus formation and the relative fluorescence at different time points are shown. Statistically analysis of FeCl3-induced thrombosis by assessing thrombus area at 8 min (n = 5). Statistical analyses were performed using unpaired two-tailed Studentbus test. *NS*, no significance; **P < 0.01. WT indicates wild-type; hACE2 indicates hACE2 transgenic. **Online Figure 9.** SARS-CoV-2 directly stimulates platelets for coagulation factor release. A and B, SARS-CoV-2 induced release of Factor V (A) and Factor XIII (B) from platelets. Washed platelets from healthy donors were incubated with or without SARS-CoV-2 (1×105 PFU) for 1 h at room temperature, and subject to ELISA assays. Thrombin at 0.025 U/mL served as the positive control. Summary data of 4 experiments using platelets from different healthy donors are presented. C, SARS-CoV-2 had no effect on platelet phosphatidylserine (PS) exposure. Washed platelets from healthy donors were incubated with or without SARS-CoV-2 (1×105 PFU) for 1 h at room temperature, and then stained with Annexin V-FITC for 30 min. PS exposure was analyzed using flow cytometry. Thrombin at 0.05 U/mL served as the positive control. Representative images and summary data are presented from 4 experiments using platelets from different healthy donors. D and E, Spike protein (2 erent healthy donor release of Factor V (D) and XIII (E) from platelets. Factor V and XIII in supernatant were assessed using commercial ELISA kits. Thrombin at 0.025 U/mL served as the positive control. Summary data of 4 experiments using platelets from different healthy donors are presented. F, Spike protein (2 d. ary data of 4 experiments using platelets after 1 h incubation. Thrombin at 0.05 U/mL served as the positive control. Representative images and summary data are presented from 4 experiments using platelets from different healthy donors. Statistical analyses were performed using One-way ANOVA, followed by Tukeylthy donors. Statistical A), (B), (C), (D), (E) and (F). *NS*, no significance; **P < 0.01. **Online Figure 10.** SARS-CoV-2 directly potentiates PF4 and inflammatory cytokines secretion from platelets. A, SARS-CoV-2 and its Spike protein stimulated PF4 release from platelets. Summary data of at 3-4 experiments using platelets from different healthy donors are presented. B, C and D, SARS-CoV-2 and its Spike protein stimulated TNF-α, IL-8 and IL-1d its Spike proteinnted. of at 3-4 experim experiments using platelets from different healthy donors are presented. Washed platelets were separated from whole blood of healthy donors and adjusted to 3edfromO in platelet-poor plasma. Platelets were then challenged with SARS-CoV-2 (1×105 PFU), Spike protein (2 . Platelets were then challenged withh. E, Increased expression of PF4 in plasma from severe and critically severe COVID-19 patients, compared with healthy donors or mild and moderated COVID-19 patients. Concentrations of PF4 were measured in plasma from healthy donors, mild and moderated COVID-19 patients and severe and critically severe COVID-19 patients (n = 5). PF4 and TNF-α, IL-8 and IL-1-19 patients and severe and critically sever commercial ELISA kits. Statistical analyses were performed using One-way ANOVA, followed by Tukeykits. Sthoc analysis in (A), (B), (C), (D) and (E). *P < 0.05; **P < 0.01. **Online Figure 11.** SARS-CoV-2 directly stimulates leukocyte-platelet aggregates (LPAs) formation. A and B, SARS-CoV-2 (A) and Spike protein (B) increased leukocyte-platelet aggregates (LPAs, CD45+CD41+), monocyte-platelet aggregates (MPAs, CD14+CD41+) and neutrophil-platelet aggregates (NPAs, CD65+CD41+). After stimulation with SARS-CoV-2 (1hil-platelet aggregates (NPAs, CD65+CD41+). After(no with different antibodies for about 30 min, red blood cells were removed and the blood samples were then subjected to flow cytometry. Summary data of 5 experiments using blood samples from different healthy donors are presented. Statistical analyses were performed using unpaired two-tailed Student are pres in (A) and (B). **P < 0.01.

## Data Availability

All data generated or analysed during this study are included in this published article (and its supplementary information files).

## Abbreviations

ACE2: Angiotensin-converting enzyme 2
TMPRSS2: Transmembrane protease serine 2
PBMCs: Peripheral blood mononuclear cells
SEM: Scanning electron microscope
TEM: Transmission electron microscopy
MPV: Mean platelet volume
PCT: Plateletcrit
PDW: Platelet distribution width
PS: Phosphatidylserine
PT: Prothrombin time
PTA: Prothrombin time activity
INR: International normalized ratio
APTT: Activated partial thromboplastin time
FDPs: Fibrinogen degradation products
NP: Nucleocapsid protein
S1: Spike protein subunit 1
S2: Spike protein subunit 2
RBD: receptor-binding domain
MAPK: Mitogen-activated protein kinase
HIV: Human immunodeficiency virus
DV: Dengue virus

## References

[1] Huang C, Wang Y, Li X, Ren L, Zhao J, Hu Y (2020). Clinical features of patients infected with 2019 novel coronavirus in Wuhan China. Lancet.

[2] Clerkin KJ, Fried JA, Raikhelkar J, Sayer G, Griffin JM, Masoumi A (2020). COVID-19 and Cardiovascular Disease. Circulation.

[3] Madjid M, Safavi-Naeini P, Solomon SD, Vardeny O. Potential Effects of coronaviruses on the Cardiovascular System: A Review. JAMA Cardiol. 2020. 10.1001/jamacardio.2020.1286 32219363

[4] Driggin E, Madhavan MV, Bikdeli B, Chuich T, Laracy J, Biondi-Zoccai G (2020). Cardiovascular considerations for patients, health care workers, and health systems during the COVID-19 pandemic. J Am Coll Cardiol.

[5] Chen N, Zhou M, Dong X, Qu J, Gong F, Han Y (2020). Epidemiological and clinical characteristics of 99 cases of 2019 novel coronavirus pneumonia in Wuhan, China: a descriptive study. Lancet.

[6] Tang N, Li D, Wang X, Sun Z. Abnormal coagulation parameters are associated with poor prognosis in patients with novel coronavirus pneumonia. J Thromb Haemost. 2020. 10.1111/jth.14768 PMC7166509 32073213

[7] Wu C, Chen X, Cai Y, Xia J, Zhou X, Xu S et al: Risk Factors associated with acute respiratory distress syndrome and death in patients with coronavirus disease 2019 Pneumonia in Wuhan, China. JAMA Intern Med 2020. 10.1001/jamainternmed.2020.0994 PMC7070509 32167524

[8] Guan WJ, Ni ZY, Hu Y, Liang WH, Ou CQ, He JX, et al. Clinical characteristics of coronavirus disease 2019 in China. N Engl J Med. 2020. 10.1056/NEJMoa2002032 PMC7092819 32109013

[9] Klok FA, Kruip M, van der Meer NJM, Arbous MS, Gommers D, Kant KM (2020). Incidence of thrombotic complications in critically ill ICU patients with COVID-19. Thromb Res.

[10] Ji X, Hou M (2011). Novel agents for anti-platelet therapy. Journal of hematology & oncology.

[11] Huang J, Li X, Shi X, Zhu M, Wang J, Huang S (2019). Platelet integrin alphaIIbbeta3: signal transduction, regulation, and its therapeutic targeting. J Hematol Oncol.

[12] Xu XR, Zhang D, Oswald BE, Carrim N, Wang X, Hou Y (2016). Platelets are versatile cells: New discoveries in hemostasis, thrombosis, immune responses, tumor metastasis and beyond. Crit Rev Clin Lab Sci.

[13] Assinger A, Kral JB, Yaiw KC, Schrottmaier WC, Kurzejamska E, Wang Y (2014). Human cytomegalovirus-platelet interaction triggers toll-like receptor 2-dependent proinflammatory and proangiogenic responses. Arterioscler Thromb Vasc Biol.

[14] Guo L, Feng K, Wang YC, Mei JJ, Ning RT, Zheng HW (2017). Critical role of CXCL4 in the lung pathogenesis of influenza (H1N1) respiratory infection. Mucosal Immunol.

[15] Chaipan C, Soilleux EJ, Simpson P, Hofmann H, Gramberg T, Marzi A (2006). DC-SIGN and CLEC-2 mediate human immunodeficiency virus type 1 capture by platelets. J Virol.

[16] Simon AY, Sutherland MR, Pryzdial EL (2015). Dengue virus binding and replication by platelets. Blood.

[17] Boilard E, Pare G, Rousseau M, Cloutier N, Dubuc I, Levesque T (2014). Influenza virus H1N1 activates platelets through FcgammaRIIA signaling and thrombin generation. Blood.

[18] Rondina MT, Brewster B, Grissom CK, Zimmerman GA, Kastendieck DH, Harris ES (2012). In vivo platelet activation in critically ill patients with primary 2009 influenza A(H1N1). Chest.

[19] Sugiyama MG, Gamage A, Zyla R, Armstrong SM, Advani S, Advani A (2016). Influenza Virus Infection Induces Platelet-Endothelial Adhesion Which Contributes to Lung Injury. J Virol.

[20] Chan JF, Yuan S, Kok KH, Chu H, Yang J, To KK (2020). A familial cluster of pneumonia associated with the 2019 novel coronavirus indicating person-to-person transmission: a study of a family cluster. Lancet.

[21] Chen W, Lan Y, Yuan X, Deng X, Li Y, Cai X (2020). Detectable 2019-nCoV viral RNA in blood is a strong indicator for the further clinical severity. Emerg Microbes Infect.

[22] Zhu N, Zhang D, Wang W, Li X, Yang B, Song J (2020). A Novel Coronavirus from patients with pneumonia in China, 2019. N Engl J Med.

[23] Zhou P, Yang XL, Wang XG, Hu B, Zhang L, Zhang W (2020). A pneumonia outbreak associated with a new coronavirus of probable bat origin. Nature.

[24] Xu X, Chen P, Wang J, Feng J, Zhou H, Li X (2020). Evolution of the novel coronavirus from the ongoing Wuhan outbreak and modeling of its spike protein for risk of human transmission. Sci China Life Sci.

[25] Wrapp D, Wang N, Corbett KS, Goldsmith JA, Hsieh CL, Abiona O, et al. Cryo-EM structure of the 2019-nCoV spike in the prefusion conformation. Science. 2020. 10.1126/science.abb2507 PMC7164637 32075877

[26] Walls AC, Park YJ, Tortorici MA, Wall A, McGuire AT, Veesler D. Structure, function, and antigenicity of the SARS-CoV-2 Spike Glycoprotein. Cell. 2020. 10.1016/j.cell.2020.02.058 PMC7102599 32155444

[27] Chen IY, Chang SC, Wu HY, Yu TC, Wei WC, Lin S (2010). Upregulation of the chemokine (C-C motif) ligand 2 via a severe acute respiratory syndrome coronavirus spike-ACE2 signaling pathway. J Virol.

[28] Lin HX, Feng Y, Wong G, Wang L, Li B, Zhao X (2008). Identification of residues in the receptor-binding domain (RBD) of the spike protein of human coronavirus NL63 that are critical for the RBD-ACE2 receptor interaction. J Gen Virol.

[29] Heurich A, Hofmann-Winkler H, Gierer S, Liepold T, Jahn O, Pohlmann S (2014). TMPRSS2 and ADAM17 cleave ACE2 differentially and only proteolysis by TMPRSS2 augments entry driven by the severe acute respiratory syndrome coronavirus spike protein. J Virol.

[30] Kuba K, Imai Y, Rao S, Gao H, Guo F, Guan B (2005). A crucial role of angiotensin converting enzyme 2 (ACE2) in SARS coronavirus-induced lung injury. Nature medicine.

[31] Wong SK, Li W, Moore MJ, Choe H, Farzan M (2004). A 193-amino acid fragment of the SARS coronavirus S protein efficiently binds angiotensin-converting enzyme 2. J Biol Chem.

[32] Zhang XH, Wang QM, Zhang JM, Feng FE, Wang FR, Chen H (2015). Desialylation is associated with apoptosis and phagocytosis of platelets in patients with prolonged isolated thrombocytopenia after allo-HSCT. Journal of hematology & oncology.

[33] Dai B, Wu P, Xue F, Yang R, Yu Z, Dai K (2016). Integrin-alphaIIbbeta3-mediated outside-in signalling activates a negative feedback pathway to suppress platelet activation. Thromb Haemost.

[34] Hu L, Chang L, Zhang Y, Zhai L, Zhang S, Qi Z (2017). Platelets Express Activated P2Y12 Receptor in Patients With Diabetes Mellitus. Circulation.

[35] Zhang S, Zhang S, Hu L, Zhai L, Xue R, Ye J (2015). Nucleotide-binding oligomerization domain 2 receptor is expressed in platelets and enhances platelet activation and thrombosis. Circulation.

[36] Zang R, Gomez Castro MF, McCune BT, Zeng Q, Rothlauf PW, Sonnek NM, et al. TMPRSS2 and TMPRSS4 promote SARS-CoV-2 infection of human small intestinal enterocytes. Science Immunol. 2020:5(47). 10.1126/sciimmunol.abc3582 PMC7285829 32404436

[37] Glowacka I, Bertram S, Herzog P, Pfefferle S, Steffen I, Muench MO (2010). Differential downregulation of ACE2 by the spike proteins of severe acute respiratory syndrome coronavirus and human coronavirus NL63. J Virol.

[38] Ho TY, Wu SL, Chen JC, Li CC, Hsiang CY (2007). Emodin blocks the SARS coronavirus spike protein and angiotensin-converting enzyme 2 interaction. Antiviral research.

[39] Qi Y, Chen W, Liang X, Xu K, Gu X, Wu F (2018). Novel antibodies against GPIbalpha inhibit pulmonary metastasis by affecting vWF-GPIbalpha interaction. J Hematol Oncol.

[40] Sauter RJ, Sauter M, Reis ES, Emschermann FN, Nording H, Ebenhoch S (2018). Functional Relevance of the Anaphylatoxin Receptor C3aR for Platelet Function and Arterial Thrombus Formation Marks an Intersection Point Between Innate Immunity and Thrombosis. Circulation.

[41] Polasky C, Wallesch M, Loyal K, Pries R, Wollenberg B. Measurement of leukocyte-platelet aggregates (LPA) by FACS: a comparative analysis. Platelets. 2020:1–6. 10.1080/09537104.2020.1732900 32098571

[42] Koupenova M, Corkrey HA, Vitseva O, Manni G, Pang CJ, Clancy L (2019). The role of platelets in mediating a response to human influenza infection. Nature communications.

[43] Koupenova M, Vitseva O, MacKay CR, Beaulieu LM, Benjamin EJ, Mick E (2014). Platelet-TLR7 mediates host survival and platelet count during viral infection in the absence of platelet-dependent thrombosis. Blood.

[44] Liu Y, Hu M, Luo D, Yue M, Wang S, Chen X (2017). Class III PI3K positively regulates platelet activation and thrombosis via PI(3)P-Directed function of NADPH oxidase. Arterioscler Thromb Vasc Biol.

[45] Liang Y, Fu Y, Qi R, Wang M, Yang N, He L (2015). Cartilage oligomeric matrix protein is a natural inhibitor of thrombin. Blood.

[46] Hu L, Fan Z, Du H, Ni R, Zhang S, Yin K (2011). BF061, a novel antiplatelet and antithrombotic agent targeting P2Y(1)(2) receptor and phosphodiesterase. Thromb Haemost.

[47] Ni H, Denis CV, Subbarao S, Degen JL, Sato TN, Hynes RO (2000). Persistence of platelet thrombus formation in arterioles of mice lacking both von Willebrand factor and fibrinogen. J Clin Invest.

[48] Huang J, Shi X, Xi W, Liu P, Long Z, Xi X (2015). Evaluation of targeting c-Src by the RGT-containing peptide as a novel antithrombotic strategy. J Hematol Oncol.

[49] Braekkan SK, Mathiesen EB, Njolstad I, Wilsgaard T, Stormer J, Hansen JB (2010). Mean platelet volume is a risk factor for venous thromboembolism: the Tromso Study, Tromso. Norway. J Thromb Haemost.

[50] Chu SG, Becker RC, Berger PB, Bhatt DL, Eikelboom JW, Konkle B (2010). Mean platelet volume as a predictor of cardiovascular risk: a systematic review and meta-analysis. J Thromb Haemost.

[51] Tao L, Zeng Q, Li J, Xu M, Wang J, Pan Y (2017). Platelet desialylation correlates with efficacy of first-line therapies for immune thrombocytopenia. Journal of hematology & oncology.

[52] Bojkova D, Klann K, Koch B, Widera M, Krause D, Ciesek S, et al. Proteomics of SARS-CoV-2-infected host cells reveals therapy targets. Nature. 2020. 10.1038/s41586-020-2332-7 PMC7616921 32408336

[53] Hoffmann M, Kleine-Weber H, Schroeder S, Kruger N, Herrler T, Erichsen S et al: SARS-CoV-2 Cell Entry Depends on ACE2 and TMPRSS2 and Is Blocked by a Clinically Proven Protease Inhibitor. Cell 2020, 181(2):271-280 e278. 10.1016/j.cell.2020.02.052 PMC7102627 32142651

[54] Hofmann H, Geier M, Marzi A, Krumbiegel M, Peipp M, Fey GH (2004). Susceptibility to SARS coronavirus S protein-driven infection correlates with expression of angiotensin converting enzyme 2 and infection can be blocked by soluble receptor. Biochem Biophys Res Commun.

[55] Deshotels MR, Xia H, Sriramula S, Lazartigues E, Filipeanu CM (2014). Angiotensin II mediates angiotensin converting enzyme type 2 internalization and degradation through an angiotensin II type I receptor-dependent mechanism. Hypertension.

[56] Lan J, Ge J, Yu J, Shan S, Zhou H, Fan S (2020). Structure of the SARS-CoV-2 spike receptor-binding domain bound to the ACE2 receptor. Nature.

[57] Mizutani T, Fukushi S, Murakami M, Hirano T, Saijo M, Kurane I (2004). Tyrosine dephosphorylation of STAT3 in SARS coronavirus-infected Vero E6 cells. FEBS Lett.

[58] Golebiewska EM, Poole AW (2015). Platelet secretion: From haemostasis to wound healing and beyond. Blood Rev.

[59] Koenen RR (2016). The prowess of platelets in immunity and inflammation. Thrombosis and haemostasis.

[60] Vanessa Monteil HK, Prado P, Hagelkrüys A, Wimmer RA, Stahl M, Leopoldi A, Garreta E, del Pozo CH, Prosper F, Romero JP, Wirnsberger G, Zhang H, Slutsky AS, Conder R, Montserrat N, Mirazimi A, Penninger JM. Inhibition of SARS-CoV-2 infections in engineered human tissues using clinical-grade soluble human ACE2. Cell. 2020. 10.1016/j.cell.2020.04.004 PMC7181998 32333836

[61] Ku Z, Ye X (2020). Salazar GTa, Zhang N, An Z: Antibody therapies for the treatment of COVID-19. Antibody Therapeutics.

[62] Klok FA, Kruip M, van der Meer NJM, Arbous MS, Gommers D, Kant KM (2020). Confirmation of the high cumulative incidence of thrombotic complications in critically ill ICU patients with COVID-19: An updated analysis. Thromb Res.

[63] Yang X, Yang Q, Wang Y, Wu Y, Xu J, Yu Y (2020). Thrombocytopenia and its association with mortality in patients with COVID-19. J Thromb Haemost.

[64] Paranjpe I, Fuster V, Lala A, Russak A, Glicksberg BS, Levin MA, et al. Association of Treatment Dose Anticoagulation with In-Hospital Survival Among Hospitalized Patients with COVID-19. J Am Coll Cardiol. 2020. 10.1016/j.jacc.2020.05.001 PMC7202841 32387623

[65] Tang N, Li D, Wang X, Sun Z (2020). Abnormal coagulation parameters are associated with poor prognosis in patients with novel coronavirus pneumonia. Journal of thrombosis and haemostasis : JTH.

[66] Hottz ED, Azevedo-Quintanilha IG, Palhinha L, Teixeira L, Barreto EA, Pao CRR, et al. Platelet activation and platelet-monocyte aggregates formation trigger tissue factor expression in severe COVID-19 patients. Blood. 2020. 10.1182/blood.2020007252 PMC7483437 32678428

[67] Poissy J, Goutay J, Caplan M, Parmentier E, Duburcq T, Lassalle F (2020). Pulmonary embolism in patients with COVID-19: awareness of an increased prevalence. Circulation.

[68] Li C, Li J, Ni H (2020). Crosstalk Between Platelets and Microbial Pathogens. Front Immunol.

[69] Assinger A (2014). Platelets and infection - an emerging role of platelets in viral infection. Front Immunol.

[70] Stewart CA, Gay CM, Ramkumar K, Cargill KR, Cardnell RJ, Nilsson MB, et al. SARS-CoV-2 infection induces EMT-like molecular changes, including ZEB1-mediated repression of the viral receptor ACE2, in lung cancer models. bioRxiv. 2020.

[71] Zaid Y, Puhm F, Allaeys I, Naya A, Oudghiri M, Khalki L et al: Platelets can contain SARS-CoV-2 RNA and are hyperactivated in COVID-19. medRxiv 2020:2020.2006.2023.20137596. 10.1161/CIRCRESAHA.120.317703 PMC7641188 32938299

[72] Liaudet L, Szabo C (2020). Blocking mineralocorticoid receptor with spironolactone may have a wide range of therapeutic actions in severe COVID-19 disease. Critical care.

[73] South AM, Tomlinson L, Edmonston D, Hiremath S, Sparks MA (2020). Controversies of renin-angiotensin system inhibition during the COVID-19 pandemic. Nat Rev Nephrol.

[74] Manne BK, Denorme F, Middleton EA, Portier I, Rowley JW, Stubben CJ, et al. Platelet Gene Expression and Function in COVID-19 Patients. Blood. 2020. 10.1182/blood.2020007214 PMC7483430 32573711

[75] Barrett TJ, Lee A, Xia Y, Lin LH, Black M, Cotzia P, et al. Biomarkers of platelet activity and vascular health associate with thrombosis and mortality in patients with COVID-19. Circulation research. 2020. 10.1161/CIRCRESAHA.120.317803 PMC7478197 32757722

[76] Comer SP, Cullivan S, Szklanna PB, Weiss L, Cullen S, Kelliher S et al: COVID-19 induces a hyperactive phenotype in circulating platelets. medRxiv 2020:2020.2007.2024.20156240. 10.1371/journal.pbio.3001109 PMC7920383 33596198

[77] Yang H, Reheman A, Chen P, Zhu G, Hynes RO, Freedman J (2006). Fibrinogen and von Willebrand factor-independent platelet aggregation in vitro and in vivo. Journal of thrombosis and haemostasis : JTH.

[78] Manne BK, Munzer P, Badolia R, Walker-Allgaier B, Campbell RA, Middleton E (2018). PDK1 governs thromboxane generation and thrombosis in platelets by regulating activation of Raf1 in the MAPK pathway. Journal of thrombosis and haemostasis : JTH.

[79] Flevaris P, Li Z, Zhang G, Zheng Y, Liu J, Du X (2009). Two distinct roles of mitogen-activated protein kinases in platelets and a novel Rac1-MAPK-dependent integrin outside-in retractile signaling pathway. Blood.

[80] Saklatvala J, Rawlinson L, Waller RJ, Sarsfield S, Lee JC, Morton LF (1996). Role for p38 mitogen-activated protein kinase in platelet aggregation caused by collagen or a thromboxane analogue. The Journal of biological chemistry.

[81] Kauskot A, Adam F, Mazharian A, Ajzenberg N, Berrou E, Bonnefoy A (2007). Involvement of the mitogen-activated protein kinase c-Jun NH2-terminal kinase 1 in thrombus formation. The Journal of biological chemistry.

[82] Versteeg HH, Heemskerk JW, Levi M, Reitsma PH (2013). New fundamentals in hemostasis. Physiological reviews.

[83] Heemskerk JW, Mattheij NJ, Cosemans JM (2013). Platelet-based coagulation: different populations, different functions. J Thromb Haemost.

[84] Pang A, Cui Y, Chen Y, Cheng N, Delaney MK, Gu M (2018). Shear-induced integrin signaling in platelet phosphatidylserine exposure, microvesicle release, and coagulation. Blood.

