# Supplementary material for: SARS-CoV-2 binds platelet ACE2 to enhance thrombosis in COVID-19
Source: J Hematol Oncol. 2020 Sep 4;13:120. doi: 10.1186/s13045-020-00954-7 (PMC7471641; doi:10.1186/s13045-020-00954-7)
Supplement: Supplementary file 1 — Additional file 1: Expanded materials and methods.. Online Table 1. Antibodies, staining chemicals and dilutions used in this study. Online Table 2. The primers used for RT-PCR. Online Table 3. Characteristics of Healthy, Non-COVID-19 patients, Mild and moderate type COVID-19 patients and Severe and critically severe type COVID-19 patients groups before propensity score matching. Online Table 4. Patient Characteristics after propensity score matching Sever and critically severe type COVID-19 patients group to healthy group (1:2), Non-COVID-19 patients group (1:1) or Mild and moderate type COVID-19 patients group (1:2). Online Table 5. Patient Characteristics after propensity score matching healthy group to Mild and moderate type COVID-19 group (1:1) or Non-COVID-19 patients group to Mild and moderate type COVID-19 group (1:2). Online Figure 1. The flowchart showing the strategy of groups enrollment. Online Figure 2. Analysis of platelet activation in COVID-19 patients after propensity score matching. A and B, Dot plot showing increased platelet t acti activation (A) and CD62P expression (B) in severe and critically severe COVID-19 patients compared with healthy donors (1:2 matching), non-COVID-19 patients (1:1 matching) or mild and moderate COVID-19 patients (1:2 matching). C and D, Dot plot showing increased PAC-1 binding (C) and CD62P expression (D) in mild and moderate COVID-19 patients compared with healthy donors (1:1 matching), non-COVID-19 patients (2:1 matching). **P < 0.01 vs control. Statistical analyses were performed using Mann-Whitney U test. *P < 0.05; **P < 0.01. Online Figure 3. SARS-CoV-2 RNA positive platelet-poor plasma enhanced platelet aggregation compared with SARS-CoV-2 RNA negative platelet-poor plasma and healthy platelet-poor plasma. Healthy platelets were incubated with healthy platelet-poor plasma (PPP), SARS-COV-2 RNA positive PPP and SARS-COV-2 RNA negative PPP from severe and critically severe COVID-19 patients at the concentration of [file 13045_2020_954_MOESM1_ESM.pdf]

SUPPLEMENTAL MATERIAL

Expanded materials and methods

Study design of patients with COVID-19

For this study, we recruited 589 patients who were suspected of COVID-19 aged from 18 to 74 years, with symptoms of fever, cough, sputum, muscle pain and other symptoms at the first affiliated hospital of Zhengzhou University, Henan Province, China and three centers of affiliated hospital of Anhui Medical University, Anhui Province, China, from January 10 through March 18 in 2020. Of these 589 patients, 422 patients were identified as SARS-CoV-2 infection by meeting one or both criteria of chest computerized tomography (CT) manifestations and reverse transcription-polymerase chain reaction (RT-PCR). The remaining 167 patients without COVID-19 served as non-COVID-19 group. We used the following inclusion and exclusion criteria to further determine the COVID-19 group and non-COVID-19 group.

For COVID-19 group, the inclusion criteria included patients with COVID-19, aged from 18 to 74 years, who were admitted to the above-mentioned hospitals in Henan and Anhui, China from Jan 10 to Mar 18, 2020. The exclusion criteria included 1, patients with incomplete data or transferred to other hospitals; 2, acute lethal organ injury (e.g., acute myocardial infarction, acute coronary syndrome, or acute pulmonary embolism,); 3, decompensated or end stage of chronic organ dysfunction (e.g., decompensated cirrhosis, decompensated chronic renal insufficiency, or severe congestive heart failure); 4, immunosuppression (acquired immune deficiency syndrome, chemotherapy, high

doses of immunosuppressive agents); 5, presence of malignancy, pregnancy or breastfeeding. After excluding, 181 COVID-19 participants were excluded, and 241 COVID-19 participants comprising 184 mild and moderate, and 57 severe, and critically severe were included.

Severity of illness was divided into mild, moderate, severe, and critically severe; and severity was determined by a team consisting of three experienced clinicians using the Chinese Clinical Guidance for COVID-19 Pneumonia Diagnosis and Treatment (7th edition). Briefly, the criteria for disease severity determination were as follows: 1, Mild cases: the clinical symptoms were mild, and there was no sign of pneumonia on imaging; 2, Moderate cases: showing fever and respiratory symptoms with radiological findings of pneumonia; 3, Severe cases: adult cases meeting any of the following criteria: (1) Respiratory distress ($\geq$ 30 breaths/ min); (2) Oxygen saturation $\leq$ 93% at rest; (3) Arterial partial pressure of oxygen (PaO_2)/fraction of inspired oxygen (FiO_2) $\leq$ 300mmHg (1 mmHg = 0.133kPa); 4, critically severe cases meeting any of the following criteria: (1) Respiratory failure and requiring mechanical ventilation; (2) Shock; (3) With other organ failure that requires ICU care.

For non-COVID-19 group, the inclusion criteria included patients without COVID-19, aged from 18 to 74 years, who were admitted to the above-mentioned hospitals in Henan and Anhui, China from Jan 10 to Mar 18, 2020, because suspected of COVID-19. The exclusion criteria are same as COVID-19 cohort. After excluding, 107 participants were excluded, and 60 participants were included.

For healthy group, 201 volunteers >18 years of age were recruited for a reproducibility study of platelet activation. Exclusion criteria included (1) history of cardiovascular disease; (2) use of medications known to affect platelet function, including non-steroidal anti-inflammatory drugs (NSAIDs), anti-histamines, and selective serotonin reuptake inhibitors, during the 2 w prior to baseline phlebotomy; (3) history of chronic kidney and liver disease, or any known hemorrhagic diathesis. After excluding, 35 participants were excluded, and 166 participants were included.

The demographic, clinical and laboratory data were extracted from the patients' medical records. The platelet and coagulation related laboratory data were collected, including platelet counts, mean platelet volume (MPV), plateletcrit (PCT), platelet distribution width (PDW), prothrombin time (PT), prothrombin time activity (PTA), international normalized ratio (INR), activated partial thromboplastin time (APTT), fibrinogen, thrombin time (TT), D-dimer, and fibrinogen degradation products (FDPs). Thrombocytopenia was defined as platelet count less than 125×10^9 per liter.

The study was approved by the Ethics Committee of the First Affiliated Hospital of Zhengzhou University (2020-KY-121) and Ethics Committee of Anhui Medical University (2020-AH-114), and complied with the Declaration of Helsinki and good clinical practice guidelines. All participants provided written informed consent.

Viral RNA detection in blood

The viral RNA in blood was detected as previously [1]. Plasma from patients was separated from the whole blood by centrifugation at 1000 g for 20 min. Viral RNA was

extracted with Nucleic Acid Isolation Kit (Da'an Gene Corporation, Cat: DA0630, Hangzhou, China) on an automatic workstation Smart 32 (Da'an Gene Corporation, China) following the guidelines. Real-time reverse transcriptional polymerase chain reaction (RT–PCR) reagent (Da'an Gene cooperation, Cat DA0930) was employed for viral detection per the protocol. In brief, two PCR primer and probe sets, which target orf1ab (FAM reporter) and N (VIC reporter) genes separately, were added in the same reaction tube. Positive and negative controls were included for each batch of detection. Samples were considered to be viral positive when either or both set(s) gave a reliable signal(s). A cycle threshold value less than 40 is interpreted as positive.

Animal studies

The wild-type C57BL/6 mice and hACE2 transgenic mice (Cat No. T037657) were purchased from Jiangsu Gempharmatech, China. Male mice were 8–10 weeks old unless otherwise stated and housed in specific pathogen-free facilities. Animal procedures were approved by the Ethical Committee of Zhengzhou University and were conducted according to the NIH Guide for the Care and Use of Laboratory Animals (NIH Publication No. 85-23, revised 1996).

Platelet transfusion studies: wild-type male mice were exposed to γ-irradiation from a 60^{Co} source (Shanghai Institute of Radiology, Fudan University) to induce thrombocytopenia with platelet counts <5% of normal after 5-6 days. Mice were anesthetized with ketamine (90 mg/kg) and xylazine (15 mg/kg), and donor platelets were isolated from wild-type or hACE2 transgenic male mice, and stained with 2 μg/ml

calcein AM (Molecular Probes, Junction City, OR, USA) for 15 min at room temperature. Total 10^9 platelets resuspended in 200 μl saline, which brought circulating platelets to a normal range were injected into the thrombocytopenic mice through the jugular vein 2 h prior to $FeCl_3$ injury.

Mice platelets were prepared as described previously [2]. Briefly, mice were anesthetized with ketamine (90 mg/kg) and xylazine (15 mg/kg), and blood was collected from the abdominal aorta of pentobarbital sodium-anesthetized mice into syringes containing 3.8% citrate (9:1). Platelet-rich plasma was filtered through a Sepharose 2B column and isolated platelets were resuspended in Tyrode's buffer.

Materials

SARS-CoV-2 Spike Protein (40589-V08B1), SARS-CoV-2 Spike Protein S1 Subunit (40591-V08H) and SARS-CoV-2 Spike Protein S2 Subunit (40590-V08B) were from Sino biological (Beijing, China). DAPI and rhodamine-labeled phalloidin were from Beyotime (Shanghai, China). ADP, thrombin, collagen and luciferase were from Chrono-Log (PA, USA). Fibrinogen and apyrase were from Sigma (St Louis, USA). Ficoll-Paque™ Plus was from GE Healthcare (Uppsala, Sweden). The PF4 (ab189573), TNF-α (ab181421), IL-8 (ab214030), Factor V (ab137976) and Factor XIII (ab108836) ELISA kits were provided by Abcam (Cambridge, MA, USA). IL-1β (HSLB00D) ELISA kit was provided by R&D (Minneapolis, MN, USA). The anti-Spike monoclonal rabbit antibody (40592-R001) targeting the receptor-binding domain (RBD) of SARS-CoV-2 with 0.11 μg/mL of IC50 on 293T-ACE2 cells infected with SARS-CoV-2 Spike

pseudovirus was from Sino biological (Beijing, China). The primary antibodies and dilutions used were listed in the **Additional file 1: Online Table 1**.

Washed platelets and peripheral blood mononuclear cells (PBMCs) isolation

All blood donors had antecubital veins allowing a clean venepuncture and denied taking any medication during the 2 weeks preceding venepuncture. Blood was drawn without stasis into siliconized vacutainers containing 1/9 v/v 3.8% sodium citrate, then centrifuged at 150 g for 20 min and the platelet-rich plasma (PRP) was collected and diluted 3-fold in ACD (75 mM sodium citrate, 39 mM citric acid and 135 mM dextrose, pH 6.5) containing 0.1 U/ml apyrase and centrifuged for 10 min at 800 g. The platelet-poor plasma was collected and the platelet pellet was then resuspended in Tyrode's buffer (137 mM NaCl, 12 mM $NaHCO_3$, 2 mM KCl, 0.34 mM Na_2HPO_4, 1 mM $MgCl_2$, 5.5 mM glucose, and 5 mM HEPES; pH 7.4) at the concentration of 3×10^8 platelets/mL. For platelet ACE2 and TMPRSS2 expression study, platelet-rich plasma prepared as previously described was filtered through a Sepharose 2B column (Sigma-Aldrich) equilibrated in Tyrode's solution to isolate platelets [2]. PBMCs were isolated within 2 h by centrifugation on a Ficoll-Paque (15 min, 800 g), then washed twice with PBS. Cell concentration was determined using Mindray (BL-2800vet, Shenzhen, China). Blood collection was approved by the Ethics Committee of Zhengzhou University and carried out according to the guidelines and regulations. Informed consents were obtained from all the volunteers.

Platelet aggregation and ATP release

Agonist-induced platelet aggregation and ATP release from dense granules were measured using Chrono-Lumiluciferin-luciferase reagent and Chrono-Log aggregometer (Chrono-Log, Havertown, PA, USA) as previously described [3]. To explore the effects of SARS-CoV-2 and its Spike protein on platelet function, SARS-CoV-2 (1×10^5 PFU [4]) or Spike protein (2 μg/mL [5, 6]) was added to platelets for indicated times before agonist-induced stimulation.

Spreading

Coverslips (20×20 mm) were coated overnight with 100 μg/mL fibrinogen in 0.1 M $NaHCO_3$ (pH 8.3) at 4 °C. 200 μL of washed platelets (2×10^7/mL) were allowed to spread on the fibrinogen-coated surfaces at 37°C for designated duration. Slides were rinsed three times with PBS, fixed, permeabilized, and stained with rhodamine-labeled phalloidin. Adherent platelets were viewed with a fluorescence microscope (Olympus BX53, Tokyo, Japan). Images were acquired using a Retiga R1 QImaging camera. The platelet-covered area was measured using ImageJ [2].

Clot retraction

Clot retraction in platelet suspension was assayed as described previously [2]. Briefly, 2 mg/mL fibrinogen was added to the platelets at a concentration of 4×10^8/mL in Tyrode's solution, and platelet suspension was dispensed in 0.3 mL aliquots into cuvettes. Clot retraction was initiated by the addition of 1 U/mL thrombin and allowed to proceed at 37°C. Clot retraction was monitored by taking photographs at indicated time points using a digital camera. Sizes of retracted clots on photographs were

quantified using NIH Image J software.

Cell culture

The human colon cell line Caco-2 (ATCC Cat No. HTB-37) and the human lung cell line Calu-3 (ATCC Cat No. HTB-55) were cultured in Minimal Essential Medium (MEM) supplemented with 10% fetal bovine serum (FBS). The human prostate cell line PC-3 (ATCC Cat No. CRL-1435) was cultured in Ham's F-12K (Kaighn's) Medium with 10% FBS. The human cervical carcinoma cell line HeLa (ATCC Cat No. CCL-2) was cultured in Dulbecco's minimal essential medium (DMEM) supplemented with 10% FBS. All cells were collected and processed for RNA and protein extraction.

SARS-CoV-2 virus preparation and incubation

SARS-CoV-2 virus was isolated from a COVID-19 patient in Shanghai (GenBank accession No. MT121215) and propagated in Vero E6 cells [7]. Supernatant from the SARS-CoV-2 infected cells was aliquoted and stored at -80°C until use. The supernatant from mock-infected cells was used as a control. For platelet function study, around 300 μL platelets were incubated with 1×10^5 PFU of SARS-CoV-2 in Tyrode’s buffer. The infectious titers have been used in other cells. All experiments involving live SARS-CoV-2 virus were performed in a biosafety level-3 (BLS-3) laboratory [4].

Reverse Transcription Polymerase Chain Reaction

Total RNA was isolated from different cells and 1 μg of RNA was reversely transcribed to cDNA using a RNA isolation kit (TaKaRa, Kyoto, Japan) and polymerase chain reaction (RT-PCR) kit (TaKaRa, Kyoto, Japan), respectively [2]. PCR reactions were

performed with specific primers in the **Additional file 1: Online Table 2**.

Flow cytometry analysis

Flow cytometry was conducted as previously described [8]. For platelet activity test in COVID-19 patients, non-COVID-19 patients and healthy volunteers, the whole blood, drawn from healthy volunteers or from patients within 24 h of admission, was collected into sterile acid-citrate-dextrose Vacutainer tubes. The first 3 mL of blood was discarded, and samples with gross hemolysis or clotting were not used. Whole blood was diluted in Tyrode's buffer (1 : 9) and then co-stained with PE-CD41 or FITC-CD41, a selective marker of platelets, and the anti-human monoclonal antibody FITC-PAC-1, or PE-CD62P, or FITC-Annexin V. Samples were then incubated at 25°C in the dark for 10 min and analyzed immediately with a flow cytometer (BD-Accuri 6, BD Biosciences, CA, USA) and selected by gating CD41 positive events.

For measurement of leukocyte-platelet aggregates, 20 μL of anticoagulant blood was transferred into a FACS tube and diluted with 80 μL of PBS at room temperature [9]. After incubation with SARS-CoV-2 (1×10^5 PFU) or Spike protein (2 μg/mL) during staining with antibodies against CD45, CD65, CD14, and CD41 for about 20 min in the dark, 1 ml RBC Lysis/Fixation Buffer (Biolegend, California, USA) was then added and incubated for about 25 min. After washing, samples were then acquired by flow cytometry (Beckman Coulter Navios, USA).

For ACE2 and TMPRSS2 surface expression, after incubated with primary antibody (ACE2, TMPRSS2 or Rabbit IgG) for 30 min at room temperature, cells (1×10^6) were

washed with PBS twice, and stained with secondary antibody for 1 h at room temperature. After washing with PBS, the cells were resuspended with 400 μL PBS for flow cytometer analysis.

For platelet activity test in SARS-CoV-2 virus or Spike protein-treated platelets, Flow cytometry was conducted as previously described [7]. Briefly, 300 μL platelets (3×10^8/mL) were challenged with SARS-CoV-2 virus (1×10^5 PFU in 300 μL) or Spike protein (2 μg/mL) for the indicated times. Platelets were diluted to final concentration of 1×10^6, then labeled with PE-CD41 or FITC-CD41, a selective marker of platelets, and FITC-PAC-1 or PE-CD62P, in Tyrode's buffer for 30 min at room temperature. Platelets were analyzed immediately with a flow cytometer (BD-Accuri 6) and selected by gating CD41 positive events. For PS exposure experiment, platelets were stained by PE-CD41 and Annexin V-FITC in Tyrode's buffer for 30 min. The samples were analyzed immediately with a flow cytometer (BD-Accuri 6). Data were analyzed using BD-Accuri 6 software and FlowJo X. The primary antibodies and dilutions used were listed in the **Additional file 1: Online Table 1**.

Electron microscopy

Sample Preparation: Platelet morphology incubated with SARS-CoV-2 for scanning electron microscope (SEM) and transmission electron microscopy (TEM) were conducted as previously described [10]. Briefly, platelets in 6-well plates were incubated with 1×10^5 PFU of SARS-CoV-2 in Tyrode's buffer at constant rotation for 30 min and 3 h at 37 °C, and washed three times with PBS to remove virus, then

subjected to SEM and TEM, respectively.

Scanning Electron Microscopy (SEM) [10]: Platelets were fixed with a 2.5% glutaraldehyde in Sorenson Phosphate buffer (0.1 M, pH 7.4) for 2 h. After fixation, platelets were washed three times in PBS (pH 7.4) for 5 min, and then fixed with 1% Osmium tetroxide in 0.1 M PBS (pH 7.4) for 1 h. Samples were rinsed, and dehydrated in a graded series of alcohol (30%, 50%, 75%, 85%, 95%, 100%), and critical point dried. The SEM procedure was completed by mounting the platelets on a specimen stub and then gold/palladium sputter coated. The samples were examined with a SU8100 scanning electron microscope (Hitachi High-Technologies, Japan).

Transmission Electron Microscopy (TEM) [11]: Platelets were fixed with a mixture of 2.0% paraformaldehyde and 2.5% glutaraldehyde in 0.1 M Sorensen's phosphate buffer (pH 7.4) for 2 h. After fixation, platelets were washed, and post-fixed with1% osmium tetroxide in 0.1 M PBS. Samples were dehydrated in a graded series of ethanol (30%, 50%, 75%, 85%, 95%, 100%), and embedded in Epon 812. Ultrathin sections (70 nm) contrasted with uranyl acetate and lead citrates were examined using a H-7650 transmission electron microscope (Hitachi High-Technologies, Japan).

Immunoblotting

Platelets were lysed with 2 × lysis buffer (50 mmol/L Tris and 150 mmol/L NaCl, pH 7.4) containing 2 × protease inhibitor and 2 × phosphatase inhibitor. Proteins were separated by sodium dodecyl sulfate-polyacrylamide gel electrophoresis, and visualized by enhanced chemiluminescence and imaged with Tanon 5200muti (Tanon

Science, Shanghai, China) after incubation with the corresponding secondary antibodies. The primary antibodies and dilutions used in these assays are listed in the **Additional file 1: Online Table 1**.

Co-immunoprecipitation

Washed platelets subjected to different treatment were lysed with equal volumes of chilled 2 × NP-40 lysis buffer (100 mM Tris-HCL pH 7.4, 300 mM NaCl, 2 mM NaF, 2% NP-40, 2 mM EDTA, and 2 × protease and phosphatase inhibitor solution) on ice for 30 min. The supernatants were then precleared using protein A/G-agarose beads for 3 h at 4°C and then centrifuged. Immunoprecipitation was carried out using anti-phospho-Ser/Thr antibody for 2 h and then incubated with protein A/G-agarose beads overnight on rocker at 4°C. The beads were then harvested and rinsed 3 times with 1 × NP-40 lysis buffer. Bead-captured ACE2 was detected by immunoblotting. The primary antibodies and dilutions used in these assays are listed in the **Additional file 1: Online Table 1**.

Confocal microscopy

Platelets were attached to Poly-L-Lysine-coated coverslip and fixed with precooled methanol for 10 min. After washing twice in phosphate-buffered saline, platelets were blocked with 10% BSA in PBS. The platelets were then incubated with the primary antibodies and appropriate secondary antibodies in 0.15% saponin. Confocal images were captured in multitracking mode on a LSM510 Meta laser-scanning confocal microscope (Carl Zeiss, Dublin, California, USA) [12]. The primary antibodies and

dilutions used in these assays are listed in the **Additional file 1: Online Table 1**.

ELISA assays

PF4, TNF-α, IL-8, IL-1β, Factor V and Factor XIII concentrations were determined using commercial ELISA kits according to manufacturer's instructions. Briefly, the supernatant was prepared by centrifuge and added into the appropriate wells with antibodies for 1 h at room temperature. The wells were then washed three times and the color was developed by adding TMB Substrate and the reaction was stopped with STOP Solution. Optical density (OD) was measured at 450 nm using a Multiskan FC Microplate Photometer (Thermo Scientific, MA, USA).

$FeCl_3$-induced thrombosis formation in mouse mesenteric arteriole

Intravital microscopy of $FeCl_3$-injured thrombus formation in mouse mesenteric arteriole was performed as described previously with minor modification [3, 13]. Briefly, after intravenous injection of 200 μg/kg Spike protein or control (saline) according to the method reported with minor modification [14], calcein-labeled platelets were injected into C57BL/6 wild-type mice by the lateral tail vein. Thrombosis was induced by 10% $FeCl_3$ for 1 min in the mesenteric arterioles within 30 min after intravenous administration of vehicle control or Spike protein and recorded with intravital microscopy for 10 min as described previously [3].

Thrombus formation under flow conditions *ex vitro*

Flow chamber assay was prepared as described previously with little modification [12]. Briefly, Thrombus formation was evaluated in a microfluidic whole-blood perfusion

assay on a fibrillar collagen matrix under arterial shear conditions (a shear rate of 1000 s^{-1}) using a Bioflux-200 system (Fluxion). Bioflux plates were coated with fibrillar collagen (100 μg/mL) overnight and blocked with 5% BSA. Wild-type and hACE2 whole blood were incubated with mepacrine (100 μM) plus Spike protein (2 μg/mL) for 30 min and perfused over fibrillar collagen-coated bioflux plates at shear force of 40 dynes/cm^2 with a Bioflux-200 system (Fluxion, South San Francisco, CA). the platelets were allowed to adhere to collagen for 5 min, and thrombus formation were visualized in real time by a fluorescence microscope (Nikon-Ti-S, Tokyo, Japan). Images were acquired with a Nikon DS-Qi1-U3 CCD camera, and the platelet-covered area was measured using Bioflux software (Fluxion, San Francisco, CA, USA).

Statistical analysis

Given the inherent differences between patients, a series of propensity score analyses were performed for following variables: age, sex, history of smoking, hypertension, diabetes mellitus, hypercholesterolemia, stroke and COPD. When matched with healthy group, variables included age, sex and history of smoking; when matched with other groups, variables included age, sex, history of smoking, hypertension, diabetes mellitus, hypercholesterolemia, stroke and COPD. The propensity scores were estimated using a logit model. Matching was conducted using 1:1 or 1:2 nearest neighbor method with a caliper width of 0.25*SDs of the logit propensity score, which yield 28 Severe and critically severe type COVID-19 patients matched with 56 healthy subjects, 37 Severe and critically severe type COVID-19 patients matched with 37 Non-COVID-19 patients, 29 Severe and critically severe type COVID-19 patients matched with 58 Mild and

moderate type COVID-19 patients, 64 Mild and moderate type COVID-19 patients matched with 64 healthy subjects, 59 non-COVID-19 subjects matched with 115 mild and moderate subjects, All matching analyses were performed using R software (version 3.6.0).

Continuous variables are expressed as the mean ± standard deviation of the mean (SD) or median (interquartile range was defined as the difference between twenty-fifth and seventy-fifth centiles) depending on data distribution, and compared using the unpaired Student's t-test or the Mann-Whitney U test, as appropriate. Categorical variables were expressed as number and percentage and compared using the Chi-square test or Fisher's exact test. Prior to statistical analysis, all data were tested for normality (Kolmogorov-Smirnov), and subjected to the Bartlett's test for homogeneity of group Variances. Group comparisons were made using One-way ANOVA, followed by Tukey's post hoc analysis or Kruskal-Wallis test with Bonferroni correction. Pearson's correlation analysis was used to investigate the relationships between two variables. A *P* value of less than 0.05 was considered statistically significant. Statistical analyses were performed using the SPSS (version 21.0) and GraphPad Prism (7.0).

References

1. Chen W, Lan Y, Yuan X, Deng X, Li Y, Cai X *et al*: Detectable 2019-nCoV viral RNA in blood is a strong indicator for the further clinical severity. *Emerg Microbes Infect* 2020, 9(1):469-473.
2. Zhang S, Zhang S, Hu L, Zhai L, Xue R, Ye J *et al*: Nucleotide-binding oligomerization domain 2 receptor is expressed in platelets and enhances platelet activation and thrombosis. *Circulation* 2015, 131(13):1160-1170.

3. Hu L, Chang L, Zhang Y, Zhai L, Zhang S, Qi Z *et al*: Platelets Express Activated P2Y12 Receptor in Patients With Diabetes Mellitus. *Circulation* 2017, 136(9):817-833.
4. Zang R, Gomez Castro MF, McCune BT, Zeng Q, Rothlauf PW, Sonnek NM *et al*: TMPRSS2 and TMPRSS4 promote SARS-CoV-2 infection of human small intestinal enterocytes. *Sci Immunol* 2020, 5(47).
5. Glowacka I, Bertram S, Herzog P, Pfefferle S, Steffen I, Muench MO *et al*: Differential downregulation of ACE2 by the spike proteins of severe acute respiratory syndrome coronavirus and human coronavirus NL63. *J Virol* 2010, 84(2):1198-1205.
6. Ho TY, Wu SL, Chen JC, Li CC, Hsiang CY: Emodin blocks the SARS coronavirus spike protein and angiotensin-converting enzyme 2 interaction. *Antiviral research* 2007, 74(2):92-101.
7. Zhou P, Yang XL, Wang XG, Hu B, Zhang L, Zhang W *et al*: A pneumonia outbreak associated with a new coronavirus of probable bat origin. *Nature* 2020, 579(7798):270-273.
8. Sauter RJ, Sauter M, Reis ES, Emschermann FN, Nording H, Ebenhoch S *et al*: Functional Relevance of the Anaphylatoxin Receptor C3aR for Platelet Function and Arterial Thrombus Formation Marks an Intersection Point Between Innate Immunity and Thrombosis. *Circulation* 2018, 138(16):1720-1735.
9. Polasky C, Wallesch M, Loyal K, Pries R, Wollenberg B: Measurement of leukocyte-platelet aggregates (LPA) by FACS: a comparative analysis. *Platelets* 2020:1-6.
10. Koupenova M, Corkrey HA, Vitseva O, Manni G, Pang CJ, Clancy L *et al*: The role of platelets in mediating a response to human influenza infection. *Nature communications* 2019, 10(1):1780.
11. Koupenova M, Vitseva O, MacKay CR, Beaulieu LM, Benjamin EJ, Mick E *et al*: Platelet-TLR7 mediates host survival and platelet count during viral infection in the absence of platelet-dependent thrombosis. *Blood* 2014, 124(5):791-802.
12. Liu Y, Hu M, Luo D, Yue M, Wang S, Chen X *et al*: Class III PI3K Positively Regulates Platelet Activation and Thrombosis via PI(3)P-Directed Function of NADPH Oxidase. *Arterioscler Thromb Vasc Biol* 2017, 37(11):2075-2086.
13. Hu L, Fan Z, Du H, Ni R, Zhang S, Yin K *et al*: BF061, a novel antiplatelet and antithrombotic agent targeting P2Y(1)(2) receptor and phosphodiesterase. *Thromb*

Haemost 2011, 106(6):1203-1214.

14. Kuba K, Imai Y, Rao S, Gao H, Guo F, Guan B *et al*: A crucial role of angiotensin converting enzyme 2 (ACE2) in SARS coronavirus-induced lung injury. *Nature medicine* 2005, 11(8):875-879.

Online Table 1. Antibodies, staining chemicals and dilutions used in this study

Antibody	Dilutions for WB	Dilutions for IP	Dilutions for FCM	Dilutions for IF	Company	Catalogue No.
Rabbit anti ACE2	1:1000		1:100		Cell Signaling Technology	4355
Rabbit anti CD14	1:1000				Abcam	ab133335
Mouse anti ACE2		1:50			R&D Systems	MAB9331
Rabbit anti-phospho-(Ser/Thr)	1:200				Abcam	ab117253
Rabbit anti ACE2				1:500	Sino Biological	10108-T60
Rabbit anti TMPRSS2	1:1000		1:100		Abcam	ab92323
Rabbit anti TMPRSS2				1:500	Invitrogen	PA5-14264
APC-Mouse anti CD41			1:100		BD Bioscience	561422
PE-Mouse anti CD62P			1:100		BD Bioscience	551142
FITC-Mouse anti PAC-1			1:100		Thermo Fisher	MA5-28564
KrO-Mouse anti CD45			1:100		Immunotech SAS	B36294
PE-Mouse anti CD41			1:100		Immunotech SAS	A07781
FITC-Mouse anti CD65			1:100		Immunotech SAS	IM1654U
PC7-Mouse anti CD14			1:100		Immunotech SAS	A22331
Rabbit anti p-Erk	1:1000				Cell Signaling Technology	4370
Rabbit anti Erk	1:1000				Cell Signaling Technology	4695
Rabbit anti p-p38	1:1000				Cell Signaling Technology	4511
Rabbit anti p38	1:1000				Cell Signaling Technology	8690
Rabbit anti p-JNK	1:1000				Cell Signaling Technology	9251
Rabbit anti JNK	1:1000				Cell Signaling Technology	9252
Mouse anti GAPDH	1:20000				Proteintech	60004
Mouse anti SARS-CoV-2 Nucleocapsid				1:500	Sino Biological	40143-MM08
Mouse anti CD41				1:500	Proteintech	24552-1-AP
Rabbit anti CD41				1:500	Invitrogen	MA1-19381

FITC Mouse IgG1, Isotype Ctrl Antibody			1:100		Biolegend	400109
PE Mouse IgG, Isotype Ctrl Antibody			1:100		Biolegend	400213
APC Mouse IgG, Isotype Ctrl Antibody			1:100		Biolegend	400119
Alexa Fluor 488 Goat anti-Rabbit IgG			1:100	1:500	Thermo Fisher	A11034
Alexa fluor 594 Goat anti-Rabbit IgG				1:500	Jackson ImunoResearch	615-585-214
Alexa Fluor 488 Goat Anti-Mouse IgG				1:500	Jackson ImunoResearch	615-545-214
Goat Anti-Rabbit IgG HRP	1:10000				Abcam	ab205718
Goat Anti-Mouse IgG HRP	1:10000				Abcam	ab6789
Annexin V-FITC			1:100		BD Bioscience	556419
DAPI			1:100		Beyotime	C1002

Abbreviation: **WB**, western blotting; **IP**, Immunoprecipitation; **FCM**, Flow Cytometry; **IF**, immunofluorescence

Online Table 2. The primers used for RT-PCR

Gene	Homo sapiens			Mouse		
	Primer 5’ → 3’	Accession No.	Product (bp)	Primer 5’ → 3’	Accession No.	Product (bp)
**ACE2**	GTGGGATGGAGTACCGACTG	NM_001371415.1	531	GGCGACAAGCACAGACTACAA	NM_001130513.1	117
	AGGATTTTCTCCACTTCTTGCTT			GCCATCTCGTTTTTCAGGACC		
**TMPRSS2**	TGAAAGCGGGTGTGAGGAGC	NM_001135099.1	359	AAGTCCTCAGGAGCACTGTGCA	NM_015775.2	116
	GGGTCAAGGTGATGCACAGT			CAGAACCTCCAAAGCAAGACAGC		
**CD14**	TCCCGGCCATCCAGAATCTA	NM_000591.4	197	TTGAACCTCCGCAACGTGTCGT	NM_009841.4	124
	AGCGAACGACAGATTGAGGG			CGCAGGAAAAGTTGAGCGAGTG		
**GAPDH**	GGAGCGAGATCCCTCCAAAAT	NM_002046.7	197	CATCACTGCCACCCAGAAGACTG	NM_001289726.1	153
	GGCTGTTGTCATACTTCTCATGG			ATGCCAGTGAGCTTCCCGTTCAG		

Online Table 3. Characteristics of Healthy, Non-COVID-19 patients, Mild and moderate type COVID-19 patients and Severe and critically severe type COVID-19 patients groups before propensity score matching

	Healthy (n=166)	Non-COVID-19 (n=60)	Mild and moderate type COVID-19 (n=184)	Severe and critically severe type COVID-19 (n=57)	*P* value
**Age, years**	49.57±14.39	46.05±14.45	44.80±14.88	58.23±14.74	<0.001
**Men**	78(46.99%)	34(56.67%)	106 (57.61%)	38 (66.67%)	0.046
**History of smoking**	31(18.67%)	25(41.67%)	63(34.24%)	23(40.35%)	<0.001
**Medical history**					
**Hypertension**	—	18(30.00%)	26(14.13%)	15(26.32%)	0.010
**Diabetes mellitus**	—	17(28.33%)	33(17.93%)	18(31.58%)	0.049
**hypercholesterolemia**	—	11(18.33%)	24(13.04%)	16(28.07%)	0.029
**Stroke**	—	8(13.33%)	8(4.35%)	7(12.28%)	0.026
**COPD**	—	3(5.00%)	4(2.17%)	2(3.51%)	0.520
**Platelet parameters**					
**Platelet count, ×10^9 per L**	229.30±54.43	229.80±60.23	194.20±66.44	108.90±70.55	<0.001* † ‡
**MPV, fL**	8.80(8.10-9.50)	8.90(8.40-10.02)	9.60(8.90-10.38)	10.70(9.90-12.30)	<0.001* † ‡
**PCT, %**	0.21(0.19-0.25)	0.22(0.17-0.25)	0.20(0.15-0.24)	0.14(0.08-0.21)	<0.001* † ‡
**PDW, fL**	16.10(15.90-16.40)	16.15(15.90-16.44)	16.26(16.00-16.41)	16.70(16.30-17.35)	0.003* † ‡
**Coagulation function**					
**PT, s**	10.20(9.80-11.80)	10.65(9.70-11.60)	10.40(9.90-11.90)	12.50(10.45-15.60)	<0.001* † ‡
**PTA, %**	106.47(89.32-111.63)	101.13(91.21-112.99)	104.04(88.40-110.30)	83.20(62.69-104.03)	<0.001* † ‡
**INR**	0.99(0.96-1.11)	1.03(0.95-1.10)	1.01(0.97-1.12)	1.16(1.01-1.40)	<0.001* † ‡
**APTT, s**	29.71±2.40	29.86±4.98	28.75±4.89	34.76±16.63	0.017* †‡
**Fibrinogen, g/L**	3.73(2.45-4.07)	3.87(2.60-4.48)	3.46(2.51-4.38)	3.75(2.25-5.25)	0.312
**TT, s**	14.90(14.60-15.60)	15.70(14.80-16.60)	16.60(13.90-17.70)	17.10(16.30-19.00)	0.005* † ‡
**D-dimer, mg/L (FEU)**	0.58(0.46-0.77)	0.61(0.22-0.73)	0.62(0.20-0.74)	1.84(0.91-5.53)	0.011* † ‡
**FDPs, ug/ml**	2.74(1.62-3.91)	2.61(0.92-4.73)	2.59(1.66-4.10)	11.41(4.56-22.91)	<0.001* † ‡

Values are mean ± SD, %, or median (interquartile range). *Significant difference ($P < 0.05$) between "Severe and critically severe type COVID-19" and "Healthy"; † Significant difference ($P < 0.05$) between "Severe and critically severe type COVID-19" and "Non-COVID-19"; ‡ Significant difference ($P < 0.05$) between "Severe and critically severe type COVID-19" and "Mild and moderate type COVID-19".

COPD: Chronic obstructive pulmonary disease, MPV: Mean platelet volume, PCT: plateletcrit, PDW: platelet distribution width, PT: Prothrombin time, PTA: Prothrombin time activity, INR: International Normalized Ratio, APTT: Activated partial thromboplastin time, TT: Thrombin time, FDPs: Fibrinogen Degradation Products

Online Table 4. Patient Characteristics after propensity score matching Severe and critically severe type COVID-19 patients group to healthy group (1:2)，Non-COVID-19 patients group (1:1) or Mild and moderate type COVID-19 patients group (1:2)

	Severe and critically severe type COVID-19 (n=28)	healthy (n=56)	*P* value	Severe and critically severe type COVID-19 (n=37)	Non-COVID-19 (n=37)	*P* value	Severe and critically severe type COVID-19 (n=29)	Mild and moderate type COVID-19 (n=58)	*P* value
**Age, years**	56.17±19.34	51.29±14.47	0.879	49.16±14.37	51.86±14.02)	0.416	56.33±14.9	50.7±16.3	0.087
**Men**	19(67.86%)	36(64.29%)	0.746	24(64.86%)	20(54.05%)	0.344	17(58.62%)	27(46.55%)	0.289
**History of smoking**	17(60.71%)	36(64.29%)	0.750	15(40.54%)	13(35.14%)	0.632	9(31.03%)	11(18.97%)	0.207
**Medical history**									
**Hypertension**	2(7.14%)	—	—	6(16.22%)	7(18.92%)	0.760	6(20.69%)	7(12.07%)	0.288
**Diabetes mellitus**	0(0%)	—	—	2(5.41%)	10(27.03%)	0.611	7(24.14%)	12(20.69%)	0.269
**hypercholesterolemia**	1(3.57%)	—	—	8(21.62%)	9(24.32%)	0.782	7(24.14%)	10(17.24%)	0.444
**Stroke**	0(0%)	—	—	1(2.70%)	2(5.41%)	1.000	0(0%)	0(0%)	—
**COPD**	0(0%)	—	—	0(0%)	0(0%)	—	0(0%)	0(0%)	—
**Platelet parameters**									
**Platelet count, ×10^9 per L**	107.37±55.84	207.59±48.12	0.012	98.96±43.17	211.5±65.28	<0.001	91.59±62.94	189.36±61.73	<0.001
**MPV, fL**	11.45(10.35-12.7)	8.45(7.80-9.20)	<0.001	10.50(10.00-12.11)	9.00(8.30-10.03)	<0.001	10.70(10.10-12.40)	9.60(9.10-10.30)	<0.001
**PCT, %**	0.16(0.09-0.25)	0.20(0.18-0.22)	0.049	0.15(0.10-0.21)	0.22(0.17-0.25)	0.002	0.14(0.09-0.19)	0.19(0.15-0.23)	0.004
**PDW, fL**	16.75(16.20-17.50)	16.10(15.90-16.30)	<0.001	16.50(16.10-17.10)	16.20(15.90-16.50)	0.003	16.60(16.20-17.02)	16.30(16.09-16.41)	0.001
**Coagulation function**									
**PT, s**	12.20(10.45-16.50)	10.50(9.65-11.60)	0.034	11.80(10.40-15.10)	10.30(9.30-11.40)	<0.001	11.50(10.40-15.20)	10.50(10.10-11.80)	0.001
**PTA, %**	85.74(58.18-103.45)	102.86(91.21-113.68)	0.029	89.32(65.43-104.04)	105.24(93.16-118.71)	<0.001	92.17(64.87-104.04)	102.86(89.32-107.72)	<0.001
**INR**	1.14(1.01-1.46)	1.01(0.95-1.10)	0.017	1.11(1.01-1.36)	1.00(0.92-1.08)	0.018	1.09 (1.01-1.37)	1.01(0.98-1.11)	0.021
**APTT, s**	33.75±12.55	29.40±2.79	0.013	33.47±11.63	28.90±4.44	0.033	34.01±13.63	28.60±3.74	0.038
**Fibrinogen, g/L**	3.59(2.18-4.83)	3.72(2.59-3.94)	0.218	3.67(2.33-5.12)	3.75(2.45-4.42)	0.888	3.67(2.25-5.06)	3.84(2.70-4.53)	0.658
**TT, s**	17.05(16.30-18.90)	14.75(14.35-15.3)	<0.001	16.90(16.30-18.50)	15.80(14.80-16.80)	0.025	17.00(16.30-19.10)	16.90(13.60-18.30)	0.036
**D-dimer, mg/L (FEU)**	1.77(0.75-4.06)	0.42(0.34-0.57)	<0.001	1.84(0.89-6.28)	0.56(0.30-0.69)	<0.001	1.84(0.92-5.34)	0.50(0.19-0.73)	<0.001
**FDPs, ug/ml**	9.94(5.55-20.96)	2.71(1.69-4.10)	<0.001	15.35(6.42-23.32)	2.50(0.73-3.86)	<0.001	11.90(5.18-22.49)	2.54(1.83-3.86)	<0.001

Values are mean ± SD, %, or median (interquartile range).

COPD: Chronic obstructive pulmonary disease, MPV: Mean platelet volume, PCT: plateletcrit, PDW: platelet distribution width, PT: Prothrombin time, PTA: Prothrombin time activity, INR: International Normalized Ratio, APTT: Activated partial thromboplastin time, TT: Thrombin time, FDPs: Fibrinogen Degradation Products

Online Table 5. Patient Characteristics after propensity score matching healthy group to Mild and moderate type COVID-19 group (1:1) or Non-COVID-19 patients group to Mild and moderate type COVID-19 group (1:2)

	healthy (n=64)	Mild and moderate type COVID-19 (n=64)	*P* value	Non-COVID-19 (n=59)	Mild and moderate type COVID-19 (n=115)	*P* value
Age, years	47.16±14.87	44.30±11.84	0.231	46.05±14.57	45.68±14.94	0.875
Men	31(48.43%)	33(51.56%)	0.724	34(57.63%)	70(60.87%)	0.680
History of smoking	19(29.69%)	15(23.44%)	0.423	24(40.68%)	46(40%)	0.931
**Medical history**						
Hypertension	—	1(1.56%)	—	10(16.95%)	18(15.65%)	0.826
Diabetes mellitus	—	1(1.56%)	—	17(28.81%)	34(29.57%)	0.918
hypercholesterolemia	—	0(0%)	—	10(16.95%)	15(13.04%)	0.487
Stroke	—	0(0%)	—	4(6.78%)	6(5.22%)	0.736
COPD	—	0(0%)	—	3(5.08%)	3(2.61%)	0.409
**Platelet parameters**						
Platelet count, $\times 10^9$ per L	211.85±47.29	183.66±73.16	0.038	206.50±48.24	195.43±69.94	0.047
MPV, fL	8.75(7.95-9.55)	9.75(8.90-10.55)	<0.001	8.90(8.40-10.00)	9.60(9.00-10.50)	<0.001
PCT, %	0.21(0.19-0.24)	0.20(0.15-0.24)	0.143	0.22(0.18-0.25)	0.20(0.14-0.24)	0.016
PDW, fL	16.10(15.90-16.30)	16.20(16.01-16.40)	0.057	16.20(15.90-16.45)	16.30(16.01-16.41)	0.146
**Coagulation function**						
PT, s	10.35(9.70-11.80)	10.40(9.90-11.25)	0.237	10.60(9.70-11.60)	11.05(10.40-13.10)	0.191
PTA, %	104.64(89.32-112.99)	104.04(94.66-110.30)	0.381	101.70(91.21-112.99)	96.74(78.47-104.04)	0.379
INR	1.00(0.95-1.11)	1.01(0.97-1.07)	0.206	1.02(0.95-1.10)	1.06(1.01-1.21)	0.591
APTT, s	28.45±2.43	29.05±3.75	0.156	28.90±3.76	28.20±3.43	0.243
Fibrinogen, g/L	3.35(2.20-3.49)	3.61(2.48-4.38)	0.134	3.92(2.68-4.48)	3.42(2.52-4.26)	0.072
TT, s	14.90(14.50-15.60)	15.95(13.80-17.60)	0.128	15.70(14.80-16.40)	16.20(13.70-17.70)	0.513
D-dimer, mg/L (FEU)	0.47(0.34-0.63)	0.52(0.22-0.66)	0.201	0.53(0.19-0.74)	0.51(0.20-0.63)	0.237
FDPs, ug/ml	2.54(1.50-3.71)	2.67(1.69-4.29)	0.218	2.61(0.90-4.69)	3.02(1.47-4.27)	0.180

Values are mean ± SD, %, or median (interquartile range).

COPD: Chronic obstructive pulmonary disease, MPV: Mean platelet volume, PCT: plateletcrit, PDW: platelet distribution width, PT: Prothrombin time, PTA: Prothrombin time activity, INR: International Normalized Ratio, APTT: Activated partial thromboplastin time, TT: Thrombin time, FDPs: Fibrinogen Degradation Products

online Figure 1

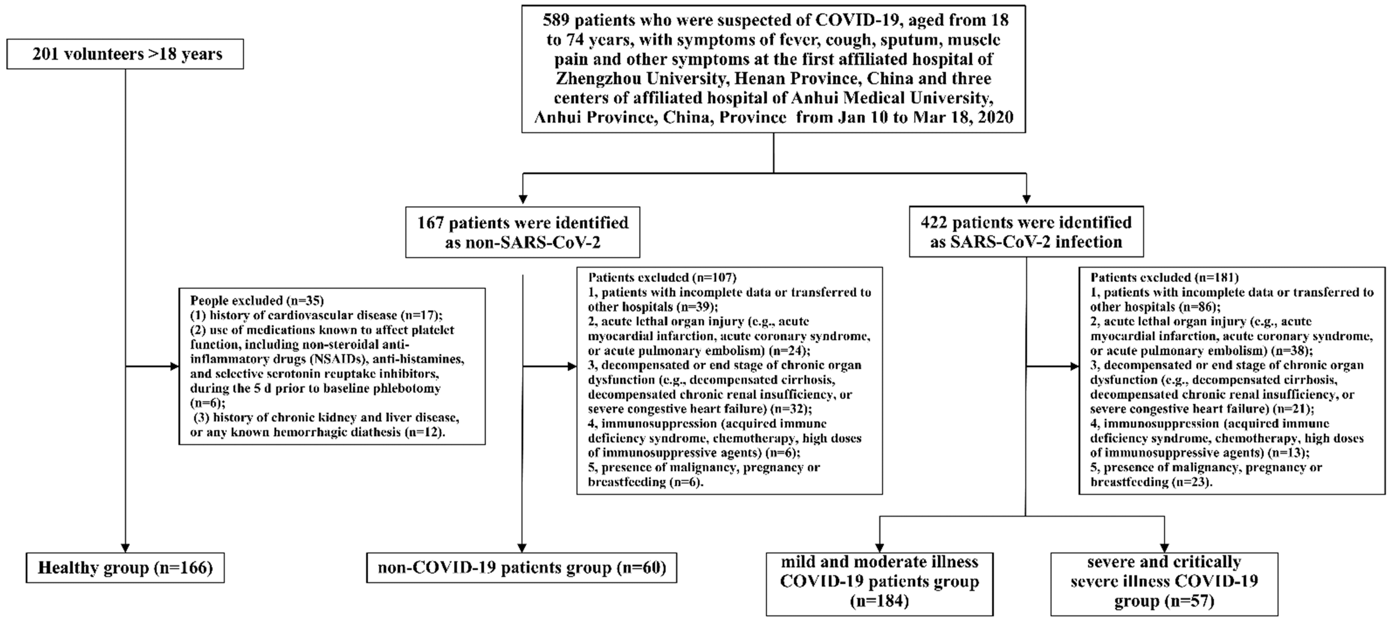

Online Figure 1. The flowchart showing the strategy of groups enrollment.

online Figure 2

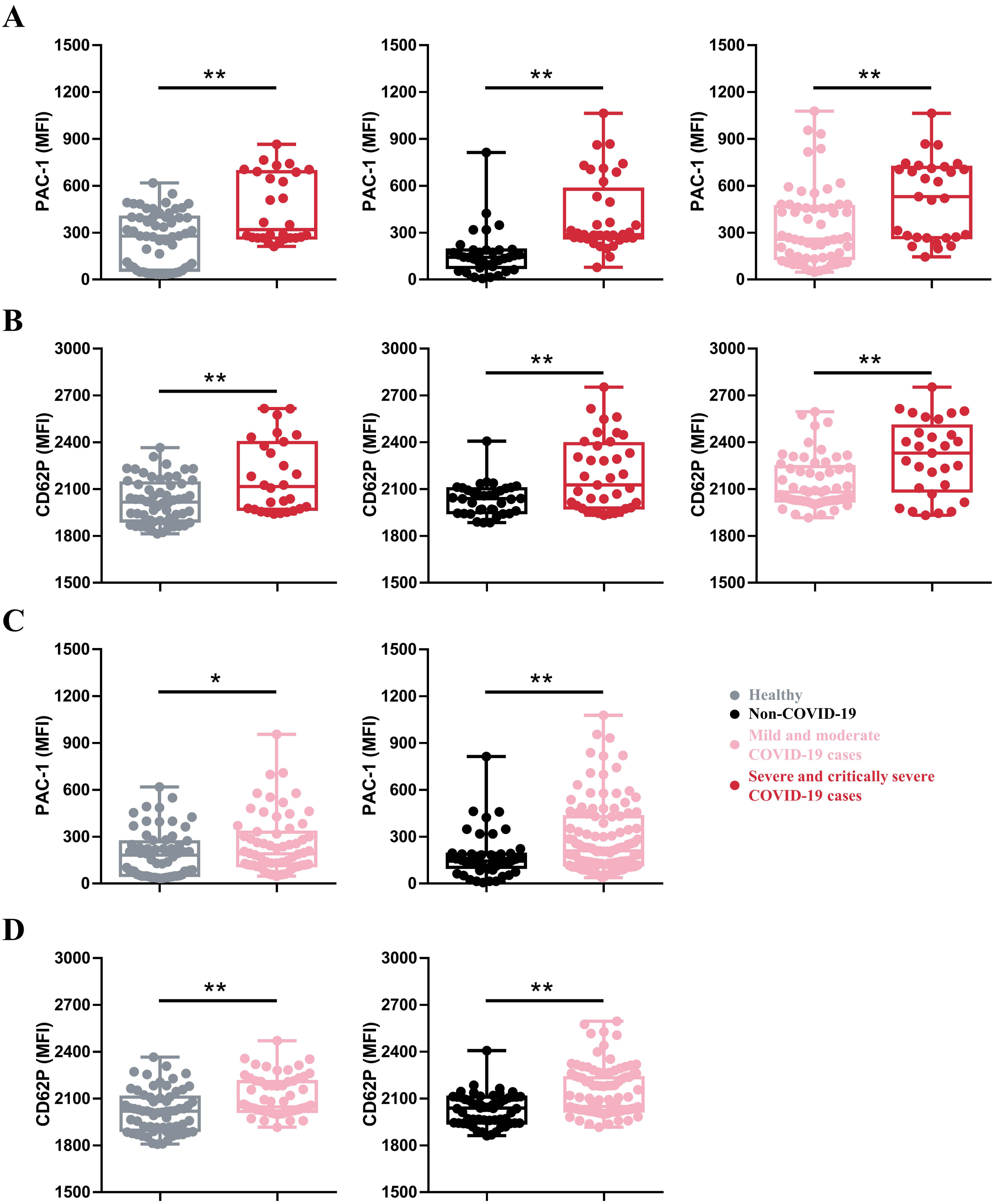

Online Figure 2. Analysis of platelet activation in COVID-19 patients after propensity score matching. A and **B,** Dot plot showing increased platelet αIIbβ3 activation (**A**) and CD62P expression (**B**) in severe and critically severe COVID-19 patients compared with healthy donors (1:2 matching), non-COVID-19 patients (1:1 matching) or mild and moderate COVID-19 patients (1:2 matching). **C** and **D**, Dot plot showing increased PAC-1 binding (**C**) and CD62P expression (**D**) in mild and moderate COVID-19 patients compared with healthy donors (1:1 matching), non-COVID-19 patients (2:1 matching). $**P < 0.01$ vs control. Statistical analyses were performed using Mann-Whitney U test. $*P < 0.05$; $**P < 0.01$.

online Figure 3

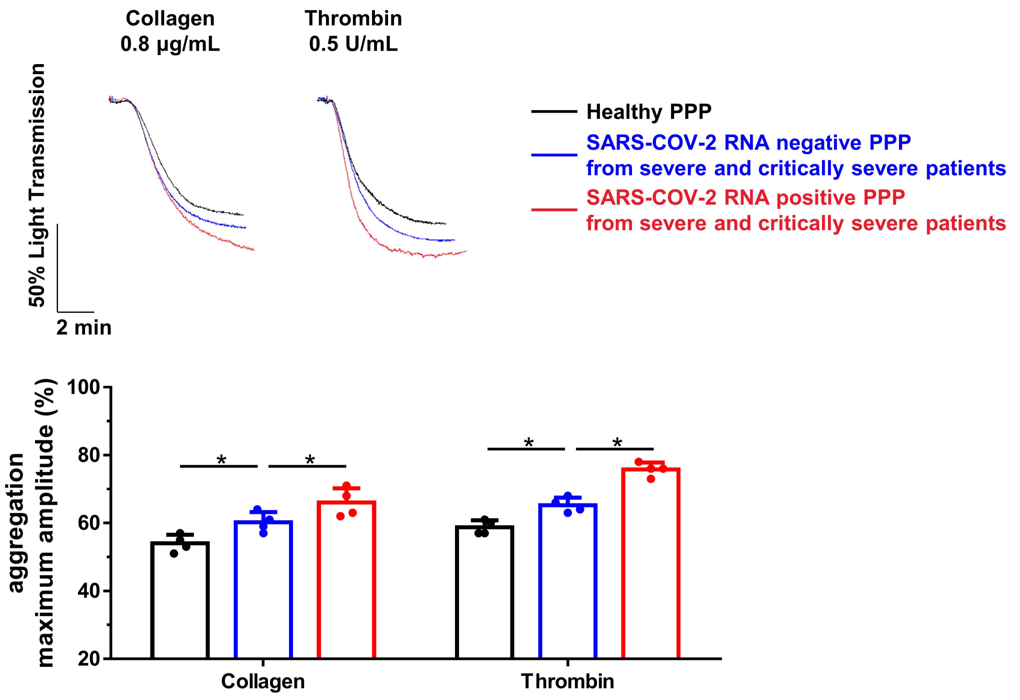

Online Figure 3. SARS-CoV-2 RNA positive platelet-poor plasma enhanced platelet aggregation compared with SARS-CoV-2 RNA negative platelet-poor plasma and healthy platelet-poor plasma. Healthy platelets were incubated with healthy platelet-poor plasma (PPP), SARS-COV-2 RNA positive PPP and SARS-COV-2 RNA negative PPP from severe and critically severe COVID-19 patients at the concentration of 2×10^8 platelets/mL for 30 min, and subjected to platelet aggregation experiment. Statistical analyses were performed using one-way ANOVA followed by Tukey's post hoc analysis. *$P < 0.05$, n = 4.

online Figure 4

SARS-CoV-2 (1×10^5 PFU)

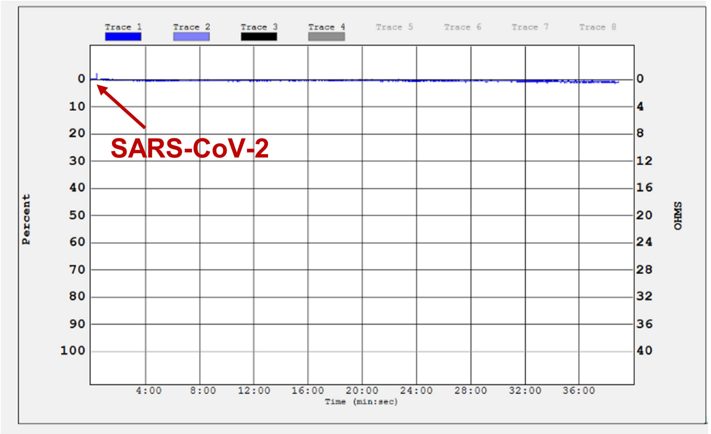

Spike (2 μg/mL)

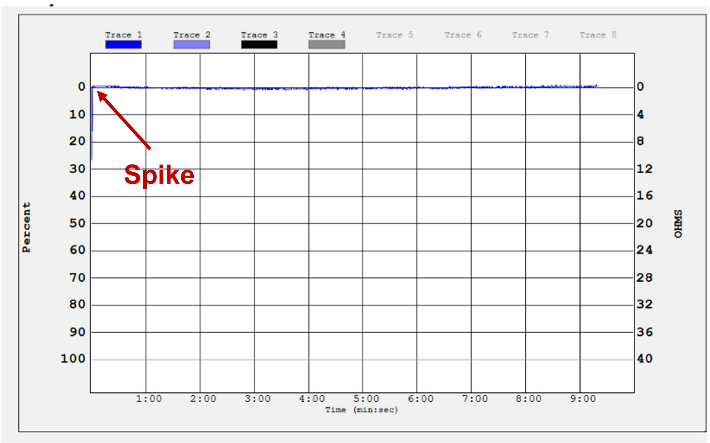

Online Figure 4. SARS-CoV-2 and its Spike protein did not induce platelet aggregation in the absence of agonist. Platelets were treated with SARS-CoV-2 ((1×10^5 PFU) or Spike protein (2 μg/mL) and platelet aggregation were monitored in indicated time in aggregometry.

online Figure 5

A

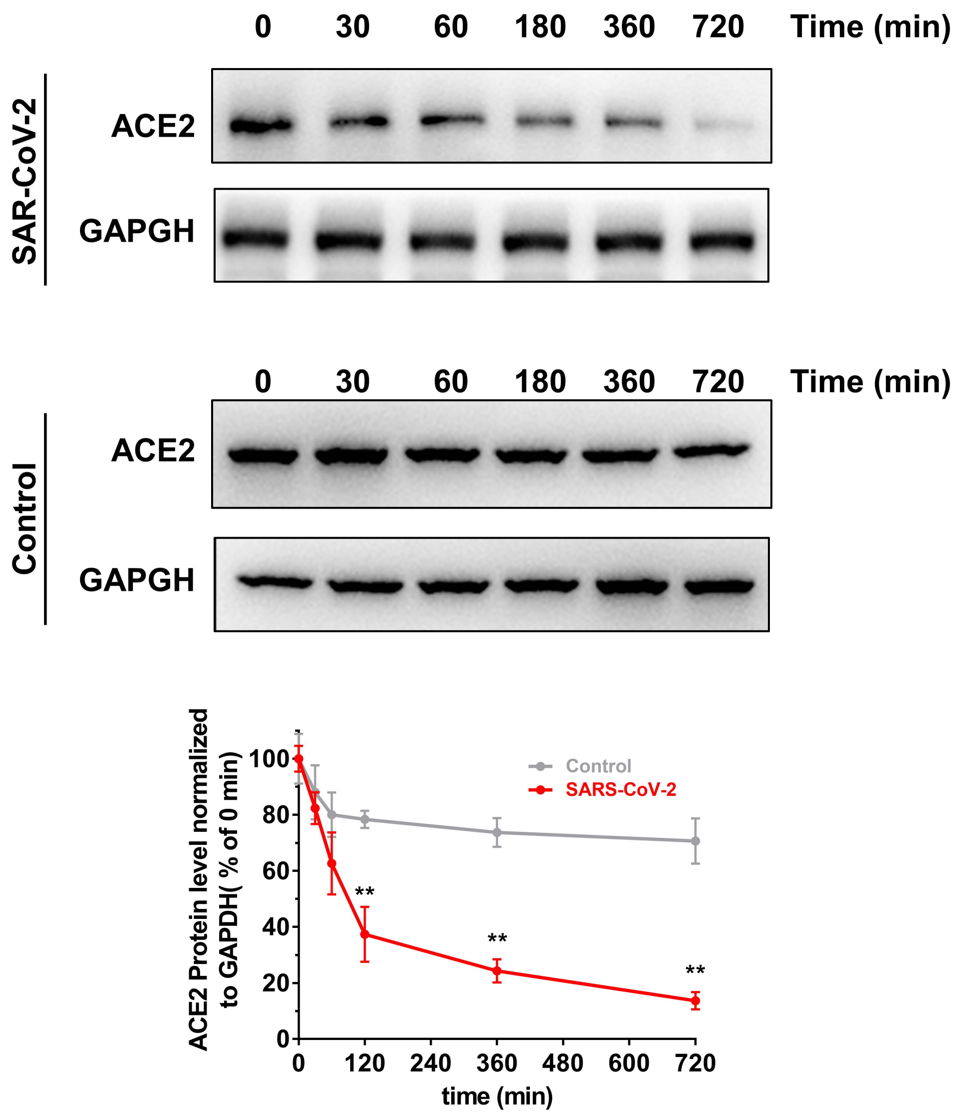

B

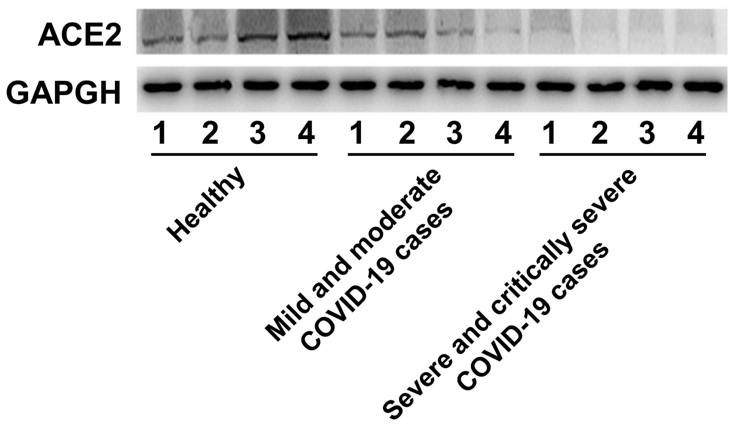

Online Figure 5. Platelet ACE2 is reduced upon SARS-CoV-2 treatment. A, Platelets from healthy donors were pretreated with SARS-CoV-2 (1×10^5 PFU) for various times, as indicated. The ACE2 protein level was detected by Western blot. Representative images and summary data of 3 experiments are presented using platelets from different healthy donors. Statistical analyses were performed using two-way ANOVA and Tukey's post hoc test. $^{**}P < 0.01$ vs control. **B**, Decreased ACE2 expression in platelets from COVID-19 patients. Representative images of 12 different individuals from healthy group (n = 4), mild and moderate COVID-19 group (n = 4) and severe and critically severe COVID-19 group (n = 4) are presented.

online Figure 6

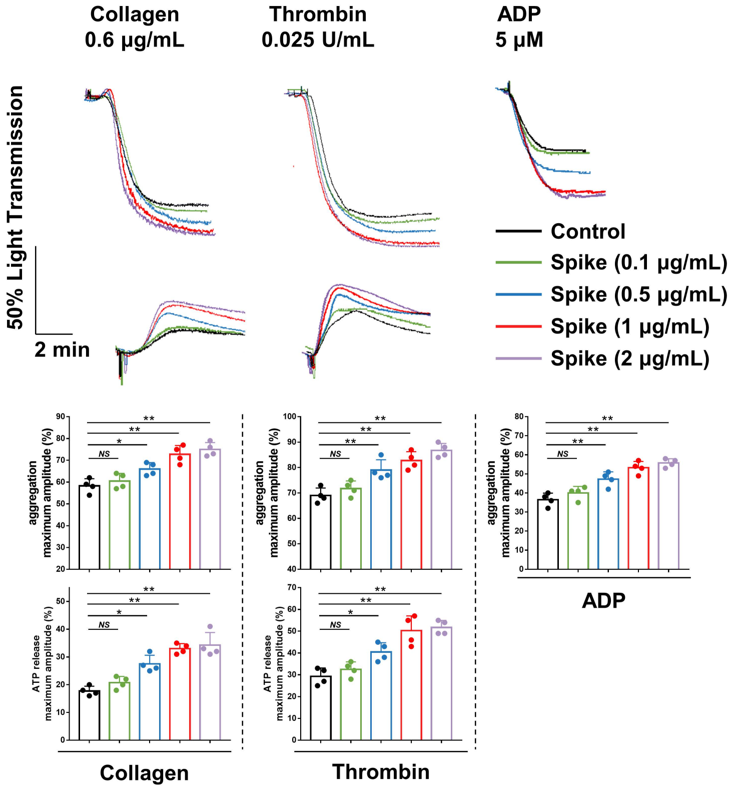

Online Figure 6. SARS-CoV-2 Spike protein dose-dependently enhance platelet activation. Washed platelets from healthy donors were incubated with Spike protein in the indicated concentration for 5 min, then stimulated with collagen (0.6 μg/mL), thrombin (0.025 U/mL), or ADP (5 μM). Aggregation and ATP release (with luciferase) were assessed under stirring at 1200 rpm. Representative results and summary data of 4 experiments are presented. Statistical analyses were performed using One-way ANOVA, followed by Tukey's post hoc analysis. NS, no significance; *$P < 0.05$; **$P < 0.01$.

online Figure 7

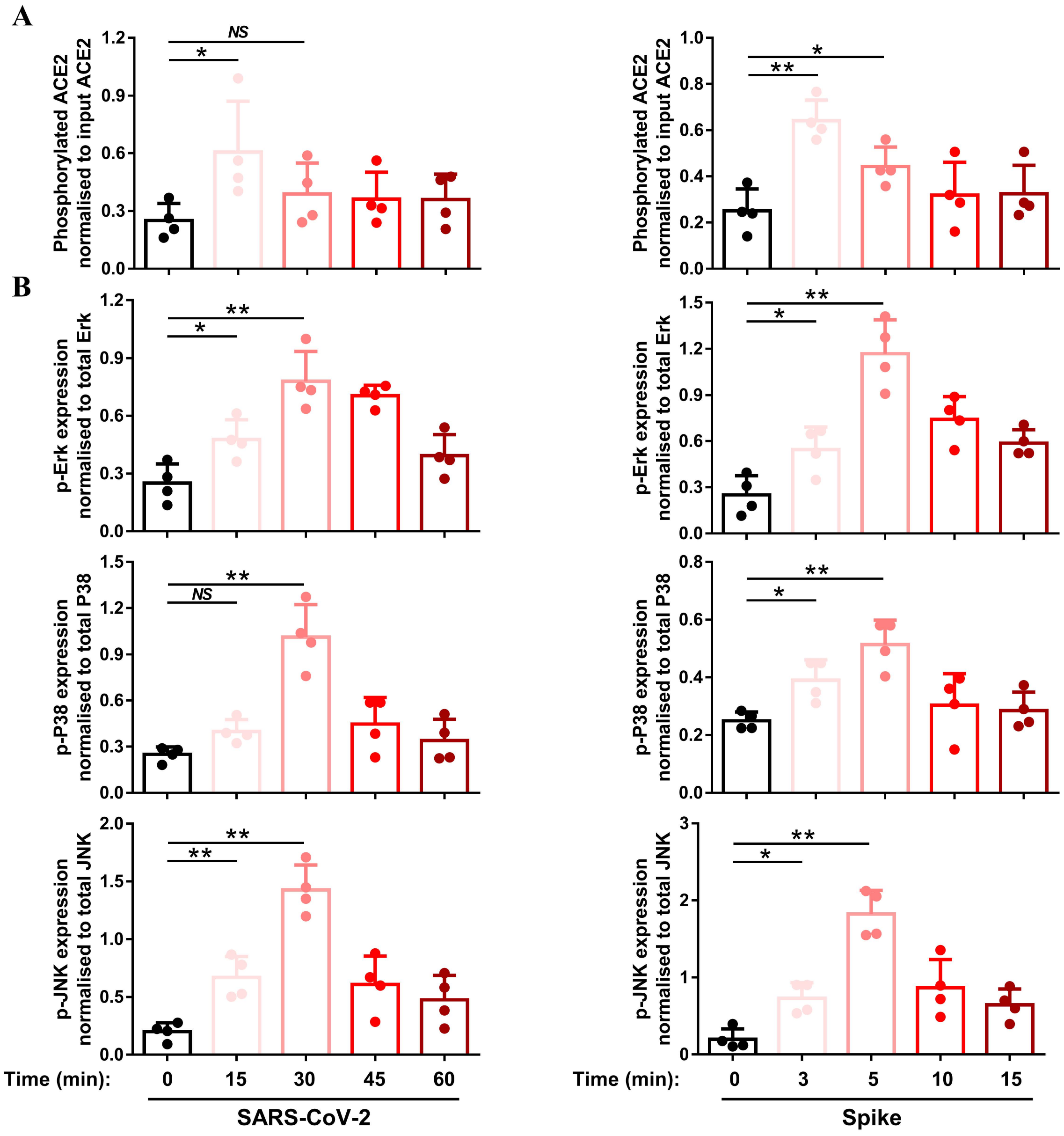

Online Figure 7. SARS-CoV-2 and its Spike protein phosphorylates ACE2, Erk, p38, and JNK in human platelets. **A**, Platelets were challenged with SARS-CoV-2 (1 x 10^5 PFU) or Spike protein (2 µg/mL), and quantitative analyses of phosphorylated ACE2 normalized to input ACE2, corresponding to **Figure 5A**, are provided from 4 experiments using platelets from different donors. **B**, Platelets were challenged with SARS-CoV-2 (1 x 10^5 PFU) or Spike protein (2 µg/mL), and quantitative analyses of p-Erk normalized to Erk, p-p38 normalized to p38, and p-JNK normalized to JNK, , corresponding to **Figure 5A**, are provided from 4 experiments using platelets from different donors. Statistical analyses were performed using One-way ANOVA, followed by Tukey's post hoc analysis in (**A**) and (**B**). *NS*, no significance; $^*P < 0.05$; $^{**}P < 0.01$.

online Figure 8

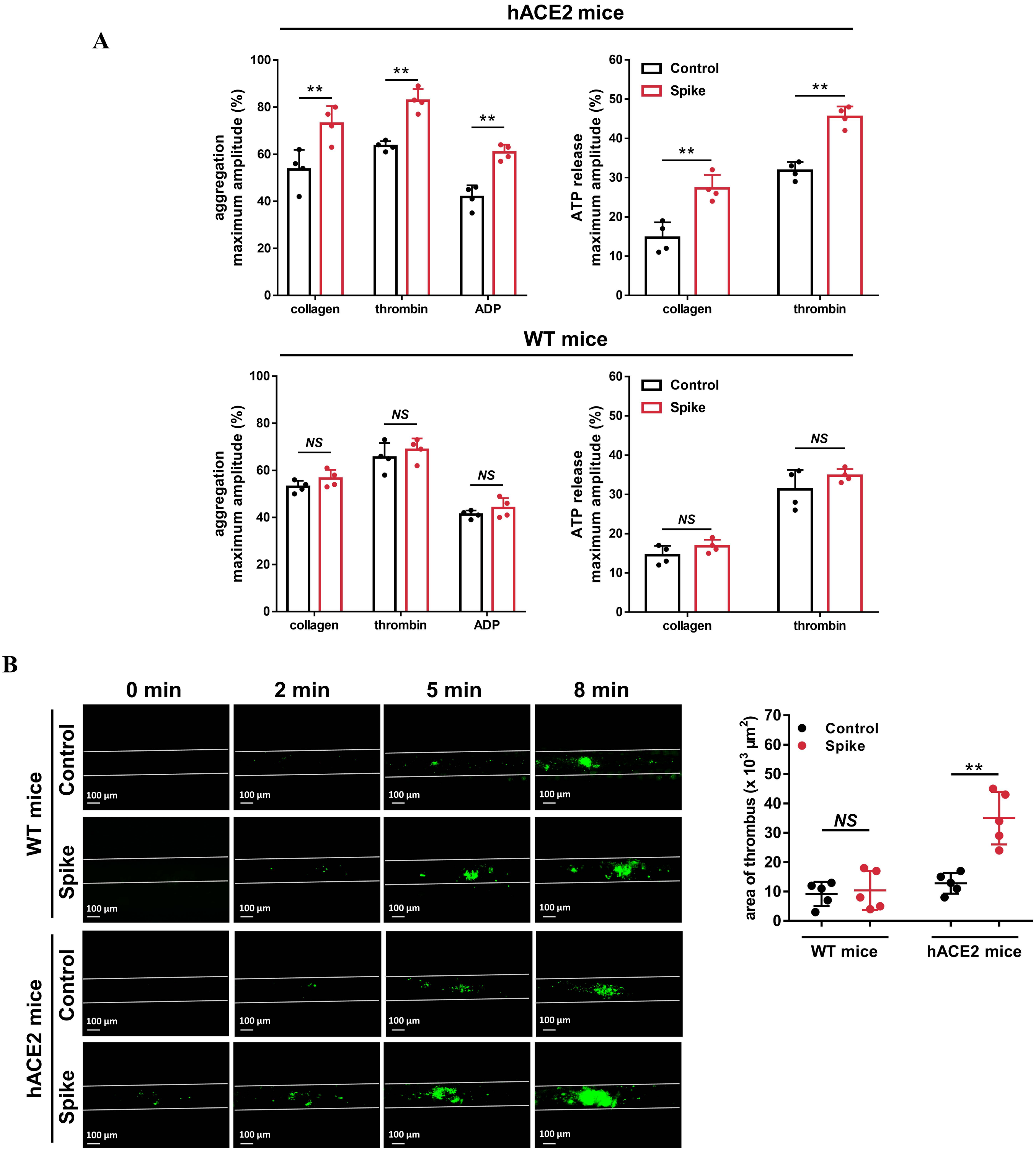

Online Figure 8. Spike protein enhances *in vitro* platelet activation and *in vivo* thrombosis potential in hACE2 transgenic mice. A, Quantitative analysis of platelets aggregation and ATP release corresponding to **Figure 6C**. Data are provided from 4 experiments using platelets from different mice. **B,** After intravenous injection 200 µg/kg Spike protein or control (saline), $FeCl_3$-induced arterial thrombus formation was initiated, and the thrombus area was recorded. Representative image of thrombus formation and the relative fluorescence at different time points are shown. Statistically analysis of $FeCl_3$-induced thrombosis by assessing thrombus area at 8 min (n = 5). Statistical analyses were performed using unpaired two-tailed Student's t test. *NS*, no significance; $^{**}P < 0.01$. WT indicates wild-type; hACE2 indicates hACE2 transgenic.

online Figure 9

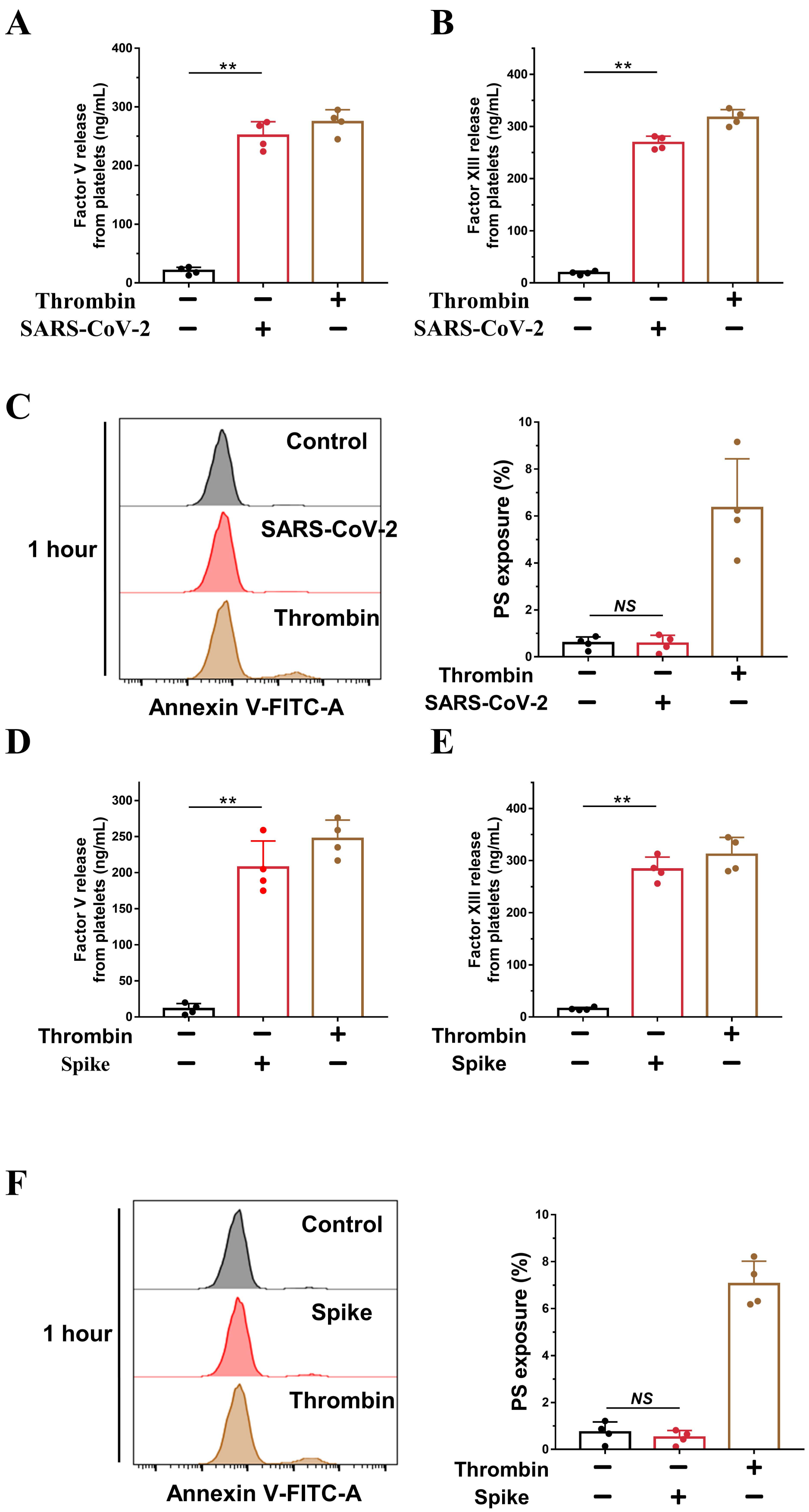

Online Figure 9. SARS-CoV-2 directly stimulates platelets for coagulation factor release. **A** and **B**, SARS-CoV-2 induced release of Factor V (**A**) and Factor XIII (**B**) from platelets. Washed platelets from healthy donors were incubated with or without SARS-CoV-2 (1×10^5 PFU) for 1 h at room temperature, and subject to ELISA assays. Thrombin at 0.025 U/mL served as the positive control. Summary data of 4 experiments using platelets from different healthy donors are presented. **C,** SARS-CoV-2 had no effect on platelet phosphatidylserine (PS) exposure. Washed platelets from healthy donors were incubated with or without SARS-CoV-2 (1×10^5 PFU) for 1 h at room temperature, and then stained with Annexin V-FITC for 30 min. PS exposure was analyzed using flow cytometry. Thrombin at 0.05 U/mL served as the positive control. Representative images and summary data are presented from 4 experiments using platelets from different healthy donors. **D** and **E**, Spike protein (2 μg/mL, 1 h) induced release of Factor V (**D**) and XIII (**E**) from platelets. Factor V and XIII in supernatant were assessed using commercial ELISA kits. Thrombin at 0.025 U/mL served as the positive control. Summary data of 4 experiments using platelets from different healthy donors are presented. **F**, Spike protein (2 μg/mL) had no effect on platelet PS exposure after 1 h incubation. Thrombin at 0.05 U/mL served as the positive control. Representative images and summary data are presented from 4 experiments using platelets from different healthy donors. Statistical analyses were performed using One-way ANOVA, followed by Tukey's post hoc analysis in (**A**), (**B**), (**C**), (**D**), (**E**) and (**F**). *NS*, no significance; $^{**}P < 0.01$.

online Figure 10

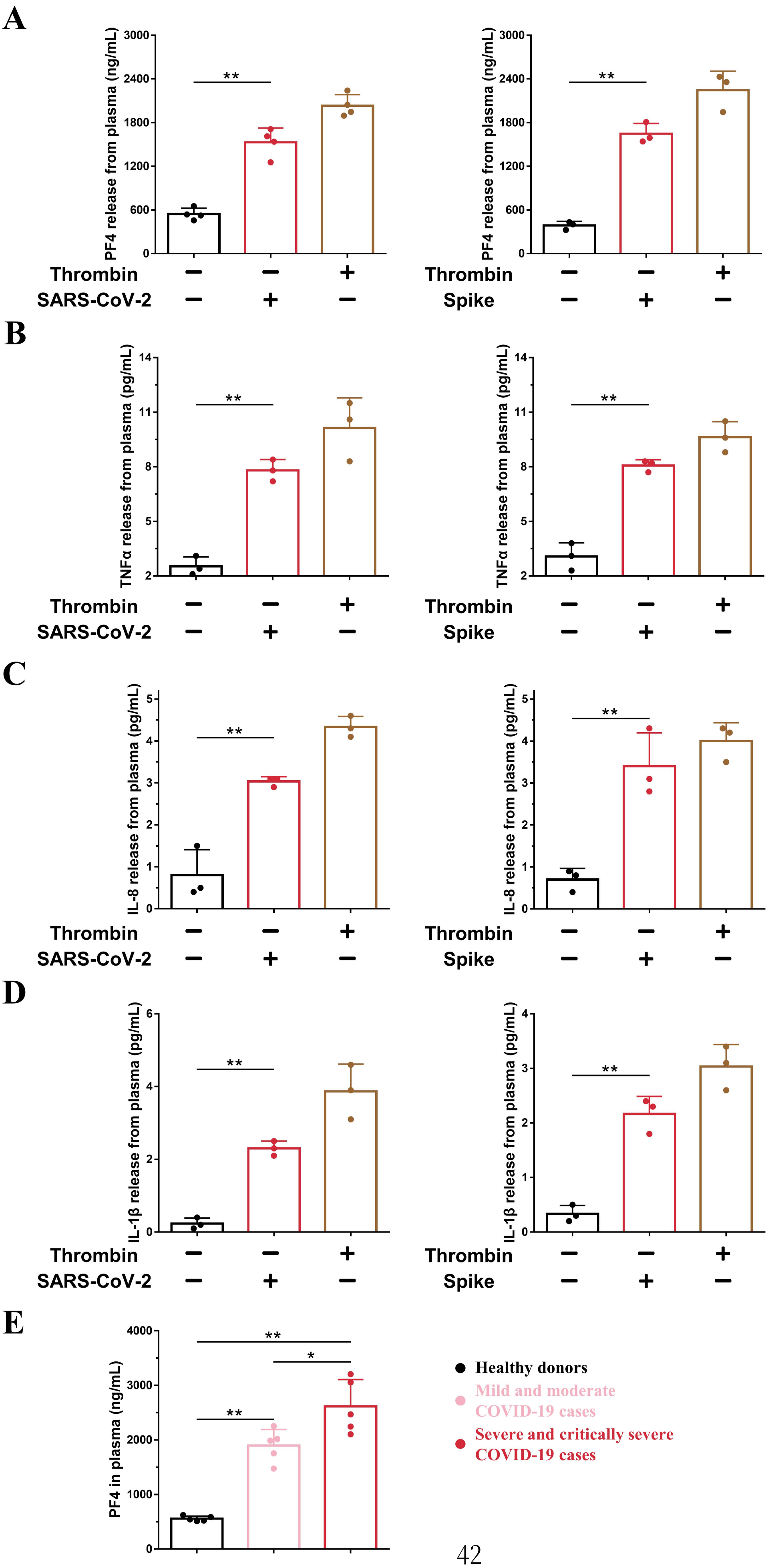

Online Figure 10. SARS-CoV-2 directly potentiates PF4 and inflammatory cytokines secretion from platelets. A, SARS-CoV-2 and its Spike protein stimulated PF4 release from platelets. Summary data of at 3-4 experiments using platelets from different healthy donors are presented. **B, C and D**, SARS-CoV-2 and its Spike protein stimulated TNF-α, IL-8 and IL-1β release from platelets. Summary data of 3 experiments using platelets from different healthy donors are presented. Washed platelets were separated from whole blood of healthy donors and adjusted to 3×10^{8}/mL in platelet-poor plasma. Platelets were then challenged with SARS-CoV-2 (1×10^{5} PFU), Spike protein (2 μg/mL) or thrombin (0.025U/mL) for 1 h. **E**, Increased expression of PF4 in plasma from severe and critically severe COVID-19 patients, compared with healthy donors or mild and moderated COVID-19 patients. Concentrations of PF4 were measured in plasma from healthy donors, mild and moderated COVID-19 patients and severe and critically severe COVID-19 patients (n = 5). PF4 and TNF-α, IL-8 and IL-1β release in supernatant were assessed using commercial ELISA kits. Statistical analyses were performed using One-way ANOVA, followed by Tukey's post hoc analysis in (**A**), (**B**), (**C**), (**D**) and (**E**). $*P < 0.05$; $**P < 0.01$.

online Figure 11

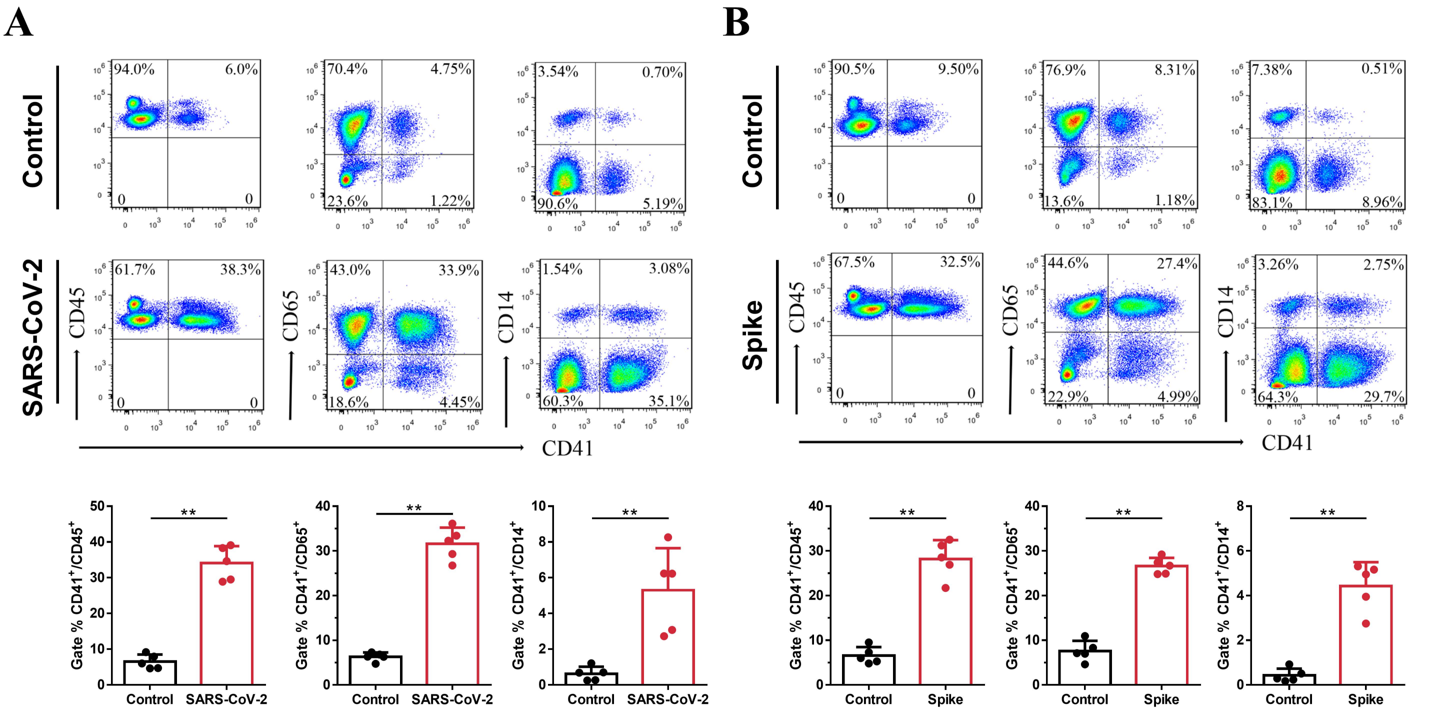

Online Figure 11. SARS-CoV-2 directly stimulates leukocyte-platelet aggregates (LPAs) formation. **A** and **B,** SARS-CoV-2 (**A**) and Spike protein (**B**) increased leukocyte-platelet aggregates (LPAs, $CD45^+CD41^+$), monocyte-platelet aggregates (MPAs, $CD14^+CD41^+$) and neutrophil-platelet aggregates (NPAs, $CD65^+CD41^+$). After stimulation with SARS-CoV-2 (1×10^5 PFU) or Spike protein (2 μg/mL) during staining with different antibodies for about 30 min, red blood cells were removed and the blood samples were then subjected to flow cytometry. Summary data of 5 experiments using blood samples from different healthy donors are presented. Statistical analyses were performed using unpaired two-tailed Student's t test in (**A**) and (**B**). $^{**}P < 0.01$.
